# Targeting metabolism by B-raf inhibitors and diclofenac restrains the viability of *BRAF*-mutated thyroid carcinomas with Hif-1α-mediated glycolytic phenotype

**DOI:** 10.1038/s41416-023-02282-2

**Published:** 2023-05-17

**Authors:** Marianna Aprile, Simona Cataldi, Caterina Perfetto, Antonio Federico, Alfredo Ciccodicola, Valerio Costa

**Affiliations:** 1 Institute of Genetics and Biophysics “Adriano Buzzati-Traverso”, CNR, Via P. Castellino 111, 80131 Naples, Italy; 2 Department of Science and Technology, University of Naples “Parthenope”, Naples, Italy; 3 Present Address: Tampere Institute for Advanced Study (IAS), Tampere University, Tampere, Finland; 4 Present Address: Finnish Hub for Development and Validation of Integrated Approaches (FHAIVE)—Faculty of Medicine and Health Technology, Tampere University, Tampere, Finland

**Keywords:** Cancer metabolism, Targeted therapies

## Abstract

**Background:**

B-raf inhibitors (BRAFi) are effective for *BRAF*-mutated papillary (PTC) and anaplastic (ATC) thyroid carcinomas, although acquired resistance impairs tumour cells’ sensitivity and/or limits drug efficacy. Targeting metabolic vulnerabilities is emerging as powerful approach in cancer.

**Methods:**

In silico analyses identified metabolic gene signatures and Hif-1α as glycolysis regulator in PTC. *BRAF*-mutated PTC, ATC and control thyroid cell lines were exposed to *HIF1A* siRNAs or chemical/drug treatments (CoCl_2_, EGF, HGF, BRAFi, MEKi and diclofenac). Genes/proteins expression, glucose uptake, lactate quantification and viability assays were used to investigate the metabolic vulnerability of *BRAF*-mutated cells.

**Results:**

A specific metabolic gene signature was identified as a hallmark of *BRAF*-mutated tumours, which display a glycolytic phenotype, characterised by enhanced glucose uptake, lactate efflux and increased expression of Hif-1α-modulated glycolytic genes. Indeed, Hif-1α stabilisation counteracts the inhibitory effects of BRAFi on these genes and on cell viability. Interestingly, targeting metabolic routes with BRAFi and diclofenac combination we could restrain the glycolytic phenotype and synergistically reduce tumour cells’ viability.

**Conclusion:**

The identification of a metabolic vulnerability of *BRAF*-mutated carcinomas and the capacity BRAFi and diclofenac combination to target metabolism open new therapeutic perspectives in maximising drug efficacy and reducing the onset of secondary resistance and drug-related toxicity.

## Background

Thyroid carcinomas, and especially the most frequent form (i.e., papillary, PTC), represent prototypic examples of how distinct oncogenic lesions drive the activation of different signalling pathways. Indeed, tumours carrying somatic mutations in *BRAF* and *RET* genes (constituting the *BRAF*-like subtype) exhibit a marked ERK-induced transcriptional signature which allows them to escape from the canonical ERK inhibitory feedback [1, 2]. On the other hand, tumours carrying either mutations in *RAS* genes or *PPARG*/PAX8 fusions (i.e., the *RAS*-like subtype) display a more pronounced induction of the PI3K/AKT signalling [1, 2]. The differential activation of signalling pathways is reflected in the lower sensitivity of *BRAF*-like tumours to the ERK-mediated inhibitory feedback loop and, from the clinical point of view, in higher tumour aggressiveness [1]. In the last decade, the alteration of distinct oncogenes has been associated with the different capacity of tumour cells to undergo metabolic reprogramming, which has emerged as a driving tumorigenesis force having a strong impact on drug responsiveness and tumour aggressiveness [3–6]. Indeed, the substantially higher glucose demand (even in normoxia) [7] is associated with increased metastatic capacity, reduced drug sensitivity [8–10] and poor prognosis [11]. Hence, identifying mutation-specific metabolic patterns is the first step to design new effective treatments to restrain tumour cells’ growth and aggressiveness.

Among the others, BRAFV600E mutation in thyroid, melanoma and colorectal carcinomas has been associated with increased expression of key glycolysis-related genes [9, 12–14]. Accordingly, B-raf inhibition by vemurafenib (VMR) in melanoma cell lines, xenograft models and primary melanomas potently suppresses glucose uptake [15–19]. Few recent studies have used The Cancer Genome Atlas (TCGA) data [1], from the thyroid carcinoma (THCA) cohort, to detect gene signatures of glucose transporters (i.e., Gluts), glycolysis and lipid metabolism genes associated with multiple clinicopathological features of PTCs [20–27]. The overexpression of specific Gluts was associated with tumour dedifferentiation [22] and increased mortality in PTC patients [20], although Gluts and glycolysis signatures were oppositely correlated to tumour differentiation [22]. Moreover, Ma et al. defined a risk score for dedifferentiation based on the expression levels of metabolic genes, which is higher in patients carrying BRAFV600E mutation [21]. Higher expression of lactate dehydrogenase A (LDHA) in PTCs in patients carrying BRAFV600E mutation has finally been related to aggressive clinicopathological features [24, 25].

Here we report—for the first time—a comprehensive investigation of all metabolic genes in TCGA-THCA cohort [1], focusing on a comparative analysis between *BRAF*- and *RAS*-like tumours. This analysis revealed subtype-specific signatures of metabolic genes in PTC, leading to the identification of Hif-1α as one the main transcriptional contributors to the metabolic rewiring of *BRAF*-driven tumours. However, among thyroid cancers, the high metastatic potential and the poorer prognosis of the anaplastic form (ATC)—of which 20–25% of cases carry BRAFV600E mutation—indicate the clinical need to design more effective therapeutic regimens to treat this aggressive tumour type. Thus, here we further report the capacity of B-raf inhibitors to restrain the glycolytic phenotype of both *BRAF*-mutated PTCs and ATCs. However, the effectiveness of B-raf inhibitors is known to decrease within 6–8 months [28–30] from starting therapy due to acquired resistance. Hence, we sought to exploit the glycolytic dependency of *BRAF*-driven tumours as a metabolic vulnerability and to adopt new therapeutic approaches to maximise drug efficacy (even at low doses) and avoid—or delay—the onset of secondary resistance and drug-related toxicity. In this regard, in line with recent reports on *BRAF*-mutated melanoma [10, 31], we found that diclofenac, a non-steroidal anti-inflammatory drug (NSAID), is able to impair the glycolytic flux of tumour cells and—synergistically with B-raf inhibitors (i.e., either vemurafenib or dabrafenib)—to reduce the viability of papillary and anaplastic thyroid cancer cells.

## Materials and methods

### TCGA data selection, processing and analysis

Whole-exome and RNA-Seq data were downloaded in March 2019 from the TCGA portal and analysed as previously described [32]. Briefly, according to the presence of specific somatic mutations, THCA patients were stratified using Exome-Seq data files (.maf format). All files retrieved from each consortium were merged. Afterwards, using a customised computational pipeline in R language, *BRAF*- and *RAS*-mutant samples were identified, and the entire cohort was subdivided into *BRAF*- and *RAS*-like tumours. Then, RNA-Seq data were used to identify patients having *RET/PTC* and *PAX8/PPARG* rearrangements to be further included in the *BRAF*-like and *RAS*-like subgroup, respectively. A total of 448 samples were analysed in this work: 327 *BRAF-*like PTCs 71 marked as *RAS-*like tumours and 50 healthy samples. The analysis of gene expression—and the identification of differentially expressed genes (DEGs)— was carried out by comparing transcriptome data (normalised values) of *BRAF*- vs *RAS*-like PTCs and of tumour vs normal samples (paired analysis). Genes differentially expressed both between the two tumour subtypes and vs healthy counterparts are indicated as *BRAF*_up_, *BRAF*_down_, *RAS*_up_ or *RAS*_down_. Differential expression analysis was carried out using generalised linear models (gLM) implemented in the EdgeR (Bioconductor package, version 3.17.10; RRID:SCR_006442; [33]). Multiple corrections were performed applying the false discovery rate (FDR) method. The FDR threshold for differentially expressed genes was set to ≤0.05. A comprehensive list of human metabolic or metabolism-related genes was retrieved in PhosphoSitePlus database http://www.phosphosite.org/psrSearchAction.do) by selecting “containing metabolism” in the section named “Protein type”, as described in ref. [34]. Moreover, the correlation analysis on gene expression data (TCGA) was performed using linear models implemented in R language (lmp function). Correlations between the selected genes were reported in the graphs as Pearson’s coefficient (*r*) and *P* values were computed by one-way ANOVA in R. DNA Methylation data (THCA) of *BRAF*- and *RAS*-mutant samples were retrieved from the TCGA repository using the TCGABiolinks (RRID:SCR_017683) Bioconductor package in March 2019 [35]. Collected data were produced by Illumina Methylation 450k microarray platform. To perform a reliable differential expression analysis, a visual inspection of probe intensity distributions was carried out, and only probes mapping in CpG islands, shores and shelves were selected for further analyses. Beta values were transformed in *M* values by applying the formula *M* = log2(Beta/(1-Beta)). Differential DNA methylation analysis between *BRAF*- and *RAS*-mutant samples was performed using the limma package in R language [36]. Nominal *P* values were adjusted by applying the Benjamini-Hochberg method [37]. Probeset IDs were annotated on the base of the *IlluminaHumanMethylation450kanno.ilmn12.hg19* annotation. Methylation data—related to probe intensities on the whole gene body—were downloaded in September 2021 from cBioportal (https://www.cbioportal.org/) for THCA cohort and were used to generate plots. Over-represented Transcription Factor Binding Site Motifs were searched using PScan online resources [38], using Jaspar 2018 matrix. Motifs’ search was carried out in a 1000-bp window upstream the transcriptional starting site of selected genes, as previously described [39]. All the heatmaps, scatter, violin and box plots were generated using Ggplot2-based scripts in R language and custom scripts, available upon request.

### Gene set enrichment analysis and LINCS L1000 data manipulation

Gene Set Enrichment Analysis (GSEA; RRID:SCR_003199) was performed by clusterProfiler R package (RRID:SCR_016884; 35). The GSEA was carried out by using the list of differentially expressed genes ranked by logFC and the KEGG pathways as gene sets. The transcriptome-wide effect of treatment with vemurafenib on cancer cell lines was evaluated by retrieving gene expression data from the L1000 Connectivity Map perturbation profiles of the Broad Institute LINCS (GEO ID: GSE70138; [36, 37]). Differential expression analysis on L1000 data for vemurafenib treatment on tumour cell lines was performed by non-parametric Wilcoxon test, comparing gene expression profiles of treated and plate-specific untreated samples as a control. All the computations carried out on the LINCS L1000 data were performed by compiling tailored R scripts. Ggplot2-based scripts in R language were used to generate the related images.

### Cell cultures

Human papillary, anaplastic thyroid carcinoma and normal thyroid follicular epithelial cell lines (i.e., BCPAP, 8505c and Nthy-ori 3-1, respectively)—kindly provided by Profs. Alfredo Fusco and Massimo Santoro (Medicina Molecolare e Biotecnologie Mediche, Università degli Studi di Napoli Federico II, Napoli, Italy)—were available in the laboratory from a previous study [40]. All cell lines—routinely analysed for Mycoplasma contamination—were “myco-free”. BCPAP were cultured in DMEM, whereas 8505c and Nthy-ori 3-1 in RPMI. Culture media were supplemented with South American foetal bovine serum (FBS, 10%), glutamine and antibiotics, and cell cultures were maintained in a humidified atmosphere of 5% CO_2_ at 37 °C. Media, sera and antibiotics were purchased from Thermo Fisher Scientific (Waltham, Massachusetts, USA). Cell lines were used for following assays between the 4th and the 16th cell passage.

### Cell treatments and multidrug combination analysis

Before treatments, all cell lines were starved for 16–18 h. For evaluating the effects of MAPK activation on metabolic genes, Nthy-ori 3-1 were treated with human recombinant Epidermal Growth Factor (EGF; 100 ng/mL; Thermo Fisher Scientific, Waltham, Massachusetts, USA) or Hepatocyte Growth Factor (HGF; 100 ng/mL; Thermo Fisher Scientific, Waltham, Massachusetts, USA) proteins for 3 or and 6 h, as previously described [40].

BCPAP were treated with PLX4032 5 µM (Selleckchem, USA, i.e., a vemurafenib analogue) at different time points: 3, 6 and 24 h for the analysis of metabolic genes and Erk phosphorylation levels, 24 h for assessing the expression levels of Hif-1α and metabolic genes—in presence or absence of Cobalt(II) chloride hexahydrate 125 µM (CoCl_2_·6H_2_O; Sigma Aldrich, St Louis, MO, USA)—and 72 h for all viability assays. Moreover, BCPAP cells were exposed for 72 h to increasing PLX4032 concentrations (from 0.5 to 25 µM) for determining LC_50_, glucose uptake and lactate secretion (0.5, 1, 5 µM). To assess Hif-1α stabilisation and the effects on metabolic genes, BCPAP were also treated with CoCl_2_ 250 µM for 24 h. Furthermore, BCPAP cells were treated with diclofenac sodium salt (Sigma Aldrich, St Louis, MO, USA; 50 µM and 100 µM) and mRNA levels of metabolic genes (3 and 6 h) and Hif-1α protein levels (24 h) have been analysed. The treatment with increasing doses of diclofenac (5, 25, 50 µM) for 72 h was performed for assessing cell viability—in presence/absence of PLX4032 (0.5, 1, 5 µM) or dabrafenib (Selleckchem, USA, 0.05, 0.1, 0.5 µM)—glucose uptake and lactate secretion.

Similarly, for viability assays 8505c cells were exposed for 72 h at PLX4032 (0.5, 1, 5, 10, 25 µM), diclofenac (5, 50, 100 µM), dabrafenib (0.05, 0.25, 0.5 µM) and trametinib (1, 5, 50 nM), alone or in combination. Particularly, for determining LC_50_ of the drugs, cells were exposed to increasing concentrations of PLX4032 (from 0.5 to 25 µM) or trametinib (MedChemExpress, USA; from 0.1 to 2000 nM). The levels of Erk phosphorylation were analysed after treatment with PLX4032 (5 and 10 µM for increasing time points from 30 min to 24 h), trametinib (50 nM for 30 min) or their combination. The expression of metabolic genes was evaluated at 3, 6 and 24 h after treatment with PLX4032 10 µM or trametinib 50 nM, whereas glucose uptake and lactate secretion were assessed 72 h after treatment with increasing doses of PLX4032 (1, 5 and 10 µM) or trametinib (1, 50 and 50 nM). The treatment of 8505c with diclofenac was also performed (100 µM for 3 and 6 h) for analysing the effects on metabolic genes, and at increasing concentrations (5, 50, 100 µM for 72 h) for evaluating glucose uptake and lactate secretion.

The effects obtained by drug combinations were assessed by analyzing viability data with SynergyFinder (https://www.synergyfinder.org/*;* RRID:SCR_019318) [41]—based on HSA reference—and by comparing the observed and expected drug combination responses (i.e., positive values denote synergy). Moreover, cNMF algorithm [42] implemented in SynergyFinder was used for the estimation of outlier measurements.

### Cell transfection

BCPAP cells were transfected with two different *HIF1A*-targeting or scrambled siRNAs (20 nM) designed from IDT (Coralville, Iowa, USA) by Lipofectamine 3000 (Life Technologies, Carlsbad, CA, USA), in culture medium without antibiotics and serum, according to manufacturer’s instructions. After 24 h, transfected cells were used for further analysis. The efficiency of Hif-1α was estimated by assessing its mRNA (by qPCR) and protein expression levels (by western blot).

### RNA extraction, RT-PCR and qPCR

Total RNA—isolated using TRIzol Reagent (Life Technologies, Carlsbad, CA, USA) as described in ref. [38]—was used for cDNA synthesis by High-Capacity cDNA Reverse Transcription kit (Invitrogen, Carlsbad, CA, USA), according to manufacturer’s instructions. Expression analysis was performed by relative qPCR assays. Gene-specific primers were designed using the Oligo 4.0 program (listed in Supplementary Table S1). The iTaq Universal Sybr Green Supermix (Bio-Rad, Hercules, CA, USA) was used for qPCR on CFX Connect Detection System (Bio-Rad, Hercules, CA, USA), according to manufacturer instructions, as previously described [39]. Relative mRNA expression was measured by the 2^-ΔΔCt^ method. The *PPIA* gene was selected as house-keeping gene and cells treated with the vehicles were used as reference samples. All reactions were performed in duplicates in at least three independent experiments. Student’s *t* test (one sample or two samples test; two-tailed) was used for assessing statistical significance of differences for normally distributed data. For each assay, SEM and statistical significance are specifically reported in figures and legends.

### Western blot

Cell lysates were extracted by RIPA buffer and blotted (30–50 mg protein/sample) with anti-Hif-1α (1:4000; Proteintech, Manchester, UK; RRID:AB_10732601), anti-pErk (1:1000; Cell Systems, Kirkland, WA, USA; RRID:AB_331646), anti-Hsp90 (1:5000; Origene, Rockville, Maryland, USA; RRID: AB_11141759), anti-Pfkfb3 (1:1000; ABclonal, Woburn, MA, USA; RRID: AB_2863158), anti-Ldha (1:1000; ABclonal, Woburn, MA, USA; RRID:AB_2758572), anti-Glut1 (1:1000; CUSABIO TECHNOLOGY LLC, Houston, USA; CSB-PA021546ESR2HU), anti-Mct4 (1:1000; CUSABIO TECHNOLOGY LLC, Houston, USA; CSB-PA021410LA01HU) antibodies and secondary anti-IgG (mouse and rabbit) antibodies (1:5000; Bio-Rad, Hercules, CA, USA; RRID: AB_11125547 and RRID: AB_2617112). The autoradiographs included in the figures are representative of at least three independent experiments. Densitometric data analysis was performed by calculating pixel density with GelQuant.NET software (http://biochemlabsolutions.com/) and displayed as mean values ± SEM of different experiments. Intensity values were normalised using the expression signals of Hsp90 and compared to signals detected in reference samples, and Student’s *t* test (two samples test; two-tailed) was used for assessing the statistical significance of differences.

### Cell viability analysis and growth curves

Viability assays were performed on BCPAP (RRID:CVCL_0153) and 8505c (RRID:CVCL_1054) cells by CellTiter-Glo Kit (Promega, Madison, Wisconsin, USA), according to the manufacturer instructions. Cells were plated and treated in 96-well white opaque plates. Luminescence was measured on VICTOR Multilabel Plate Reader (Perkin Elmer, USA). The effect of each treatment on cell viability was estimated after 72 h as the percentage of luminescence (means ± SEM of four independent experiments) compared to control cells, as specified in figure legends. The luminescence of control cells was set to 100% and Student’s *t* test (one sample or two samples test; two-tailed) was used for assessing statistical significance of differences. Moreover, for each cell line and treatment, cell growth curves were obtained using a standard curve of cell number and plotting viability values at different time points (i.e., 0 h, 12 h, 24 h, 48 h, 72 h). Then, the area under the curves (AUC) has been calculated as described in ref. [10].

### Glucose uptake and lactate secretion assays

Glucose uptake analysis was performed on Nthy-ori 3-1, BCPAP and 8505c cells using the Glucose Uptake Colorimetric Assay Kit (Sigma Aldrich, St Louis, MO, USA), according to manufacturer instructions. Cells were plated in 96-well plates, and the effect of each treatment/co-treatment was estimated after 72 h. Cells were serum-starved for 14 h and then glucose-starved for 40 min by KRPH buffer 2% BSA. Insulin (1 mM) stimulation was performed for 40 min and a negative control was prepared by incubating a parallel sample without insulin and 2-DG. Colorimetric detection of 2-DG6P was performed on VICTOR Multilabel Plate Reader (Perkin Elmer, USA). OD values were corrected for dilution factor and by subtracting background (blank sample) and negative control values. Standard curves were plotted using 2-DG6P standards. For the analysis of the total glucose uptake, each value (pmol) was normalised for the related AUC and plotted. The insulin-induced glucose uptake was calculated for each sample as the difference between insulin-stimulated and insulin-free cells. Student’s *t* test (one sample or two samples test; two-tailed) was used for assessing the statistical significance of differences.

L-lactic acid content in cell culture supernatant was measured by L-lactic acid/lactate (LA) Colorimetric Assay Kit (Elabscience, USA), according to manufacturer instructions. The supernatant samples were collected from Nthy-ori 3-1, BCPAP and 8505c plated in 96-well plates, treated or not for 72 h and centrifuged (at 10,000×*g* for 5 min, 4 °C). Colorimetric detection of secreted lactate was performed on VICTOR Multilabel Plate Reader (Perkin Elmer, USA). OD values were corrected considering background (blank sample), the dilution factor and standard concentration (mmol/L). Lactate concentration (mmol/L) was calculated by using standard curves, the obtained values were normalised for the related AUC and plotted. The effect of each treatment was estimated as the percentage of lactate secreted by control cells (set to 100%) and Student’s *t* test (one sample or two samples test; two-tailed) was used for assessing statistical significance of differences.

## Results

### Identification of subtype-specific metabolic gene signatures in *BRAF*-like PTCs

**Fig. 1:**
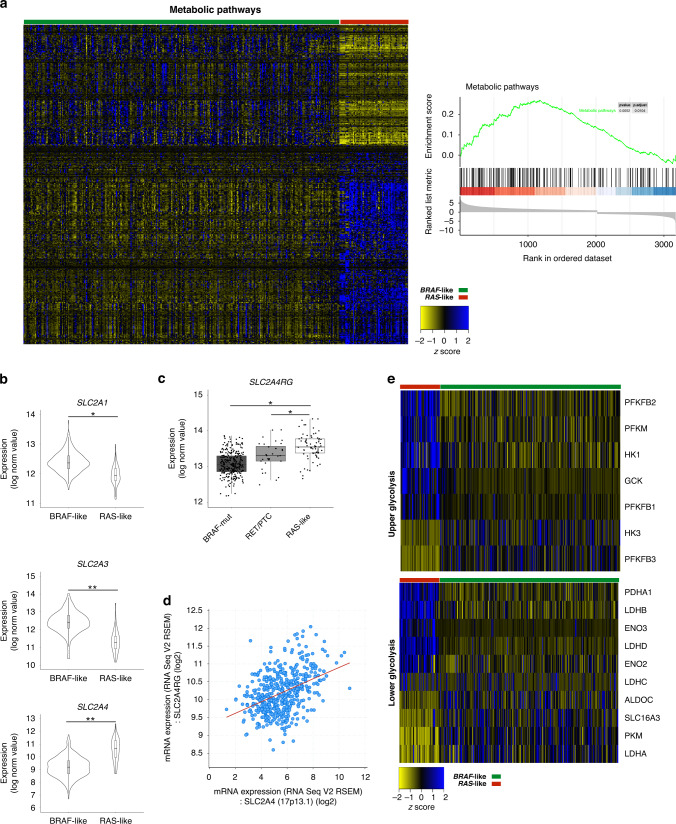
*BRAF-*like PTC subtype displays a specific signature of metabolic genes. **a** Left panel. Heatmap showing normalised expression data (RNA-Seq from THCA; *n* = 398) of genes flagged as “metabolism” or “metabolism-related” (PhosphoSitePlus database) in *BRAF*- (green) vs *RAS*-like (red) PTCs. Right panel. The enrichment score curve (in green) of genes belonging to the “Metabolic pathway” obtained as output of the Gene Set Enrichment Analysis (GSEA) on the genes differentially expressed (*P* value=0.0052; *P*-adjusted=0.0104) between *BRAF*- and *RAS*-like tumours (THCA cohort, TCGA). The vertical black lines indicate gene position in the ranked list, whereas horizontal bars in graded colour (upregulated in red; downregulated in blue) indicate the rank-ordered, non-redundant list of genes. **b** Violin plots illustrating normalised expression values (log_2_ scale) of *SLC2A1*, *SLC2A3* and *SLC2A4* – encoding glucose transporters —in *BRAF*- (*n* = 327) and *RAS*-like (*n* = 71) PTCs (THCA cohort). *FDR ≤ 0.05, **FDR ≤ 0.01, ***FDR ≤ 0.001. **c** Box plots displaying normalised expression values (log_2_ scale) of *SLC2A4RG*—encoding the main transcription regulator of Glut4—in *BRAF*-like (BRAFV600E and *RET/PTC*) and *RAS*-like tumours. *FDR ≤ 0.05. **d** Scatter plot of normalised expression data (log_2_ scale) showing the correlation trend between *SLC2A4* and *SLC2A4RG* overall THCA cohort (generated in cBioPortal); Spearman’s correlation: 0.39 (*P* = 3.98^e-19^), Pearson’s correlation: 0.41 (*P* = 3.01^e-21^). **e** Heatmaps showing normalised expression data of genes involved in upper and lower glycolysis in the *BRAF*- (green) vs *RAS*-like (red) PTCs.

To investigate whether *BRAF*-like papillary thyroid carcinomas display specific expression signatures of metabolic genes, we took advantage of TCGA whole-exome and transcriptome data to stratify tumours in the *BRAF*- and *RAS*-like subtypes and to identify differentially expressed genes (DEGs). The overall comparison of expression data from the two distinct PTC subtypes explored by GSEA indicated “Metabolic pathways” as the only enriched process (Fig. 1a). Accordingly, metabolic and metabolism-related genes rank among the top DEGs (~31%, i.e., 459/1462, gene IDs retrieved in March 2019 from PhosphoSitePlus database) and their clustering is evident between the two PTC subtypes (Fig. 1a and Supplementary File S1). Using the KEGG database, we focused on four main processes, i.e., carbohydrate, lipid, amino acid and nucleotide metabolism, which include 246 DEGs (Supplementary Fig. S1). To identify those specifically altered in distinct PTC subtypes, we selected genes differentially expressed between *BRAF*- and *RAS*-like PTCs and deregulated even vs the related healthy samples (i.e., 133 genes) and we labelled them *BRAF*_up_ and *BRAF*_down_ or *RAS*_up_ and *RAS*_down_ genes (Supplementary Fig. S1A and Supplementary File S1). In addition, taking advantage of RNA-Seq data from our previously published [2] independent cohort of PTC patients (*n* = 22), we validated ~55% of them (i.e., 73/133 genes) as differentially expressed between tumour subgroups (Supplementary File S1). Notably, *BRAF*-like tumours displayed major expression changes in several genes accounting for energy metabolism (i.e., 116 genes), whereas only 20 were identified as altered in *RAS*-like PTCs. Particularly, focusing on the expression of membrane glucose transporters, we observed that, despite *SLC2A1* gene (alias Glut1) is reported as the most abundant channel expressed in primary thyroid tumours and cell lines [43, 44], *SLC2A3* gene (alias Glut3) display comparable expression levels and both genes show higher expression in *BRAF*- vs *RAS*-like tumours (Supplementary Fig. S1B), as confirmed by RNA-Seq data of healthy thyroids on GTEx database (Supplementary Fig. S1C). However, both *SLC2A1* (FDR < 0.01) and *SLC2A3* (FDR < 0.001) genes—associated with higher glucose uptake and oncogenic growth [12, 45, 46]—are markedly overexpressed in *BRAF*- (vs *RAS*)-like tumours (Fig. 1b). Differently from *BRAF*-like PTCs, *RAS*-like tumours show higher levels of the inducible transporter *SLC2A4* (*alias* Glut4; Fig. 1b) and of its main regulator, the transcription factor *SLC2A4RG* (Fig. 1c), whose expression levels are positively correlated across tumour samples (Fig. 1d). Then, considering the processes following glucose internalisation and phosphorylation we focused on pentose phosphate pathway (PPP) and glycolysis. Except for *TKT* gene, all PPP-related genes are not differentially expressed, whereas the expression of genes encoding enzymes of upper and lower glycolysis is largely affected between tumour subtypes, also compared to the healthy counterparts (Fig. 1e). In particular, we identified a specific signature of glycolysis-related genes in *BRAF*-like tumours, which display the overexpression of *SLC2A1*, *SLC2A3*, *HK3*, *PFKFB3*, *PKM*, *LDHA* and *SLC16A3* (alias Mct4, the membrane channel regulating lactate efflux). Notably, also *LDHA/LDHB* expression ratio—measured within each tumour sample—shows a marked increase only in *BRAF*-like tumours, whereas *LDHB* is modestly upregulated in the *RAS*-like subtype (Supplementary Fig. S2A, S2B). Thus, in contrast with the glycolytic phenotype of *BRAF*-like PTCs, *RAS*-like tumours display expression signatures compatible with oxidative metabolism [47], characterised by the usage of extracellular lactate to produce ATP (via OXPHOS). Conversely, the expression of *SLC16A1* and *SLC16A7* genes (Supplementary Fig. S2C, D)—encoding MCT1 and MCT2 lactate transporters, respectively—as well as of *GJA1* (data not shown) encoding connexin 43 and responsible of lactate diffusion [48], does not differ between the two tumour subtypes. Thus, according to the flux control coefficients reported by Tanner and colleagues [49], transcriptome data indicate that *BRAF*-like tumours have a marked increase of the entire glycolytic flux—from glucose uptake to lactic fermentation and excretion—compared to *RAS*-like sample (Supplementary Fig. S3). Moreover, we also found that TCA cycle, FAO and OXPHOS genes display a subtype-specific expression pattern (Supplementary Figs. S1A and S4). The overexpression in *BRAF*-like tumours of specific genes encoding enzymes responsible for anaplerotic TCA cycle reactions (Supplementary Fig. S4A, B and Supplementary File S1) possibly contributes to replenishing the oxaloacetate pool from pyruvate and aspartate. Glutamine influx is another anaplerotic process enhanced in *BRAF*-like PTCs, which may contribute to replenishing TCA intermediates. Indeed, glutamine transporter (*SLC16A5)* and enzymes converting glutamine to glutamate (*GLS* and *GLS2*) are specifically overexpressed in *BRAF*-like PTCs (Supplementary Fig. S4A, B). Moreover, *GOT1*—regulating one of the main cataplerotic reactions—is downregulated only in this subtype (Supplementary Fig. S4A, B). In line with the high glycolytic flux and low amount of intermediates feeding the TCA cycle, *BRAF*-like tumours display a mild downregulation of OXPHOS genes compared both to *RAS*-like and healthy samples (Supplementary Fig. S4C, D and Supplementary File S1). Taken together, transcriptome data indicate that *BRAF*-like PTCs undergo extensive metabolic rewiring with subtype-specific expression patterns indicative of enhanced glucose uptake and glycolytic flux and reduced ATP production via OXPHOS. Thus, the subtype-specific expression patterns of metabolic genes strongly suggest a differential metabolic rewiring between subtypes in line with the lower thyroid differentiation score (TDS [1]) of *BRAF*-like tumours (TDS markers are reported in Supplementary Fig. S4E). Finally, confirming the subtype-specific reprogramming of metabolic genes we further disclosed that *BRAF*-like PTCs display specific patterns of expression also for genes involved in amino acid metabolism (Supplementary Fig. S5A, B), sterol and lipid biosynthesis (Supplementary Fig. S5C, D), purine and pyrimidine biosynthesis (Supplementary Fig. S6A, B).

### Analysis of DNA methylation and transcription factors as contributors to the metabolic reprogramming of *BRAF*-like PTCs

**Fig. 2:**
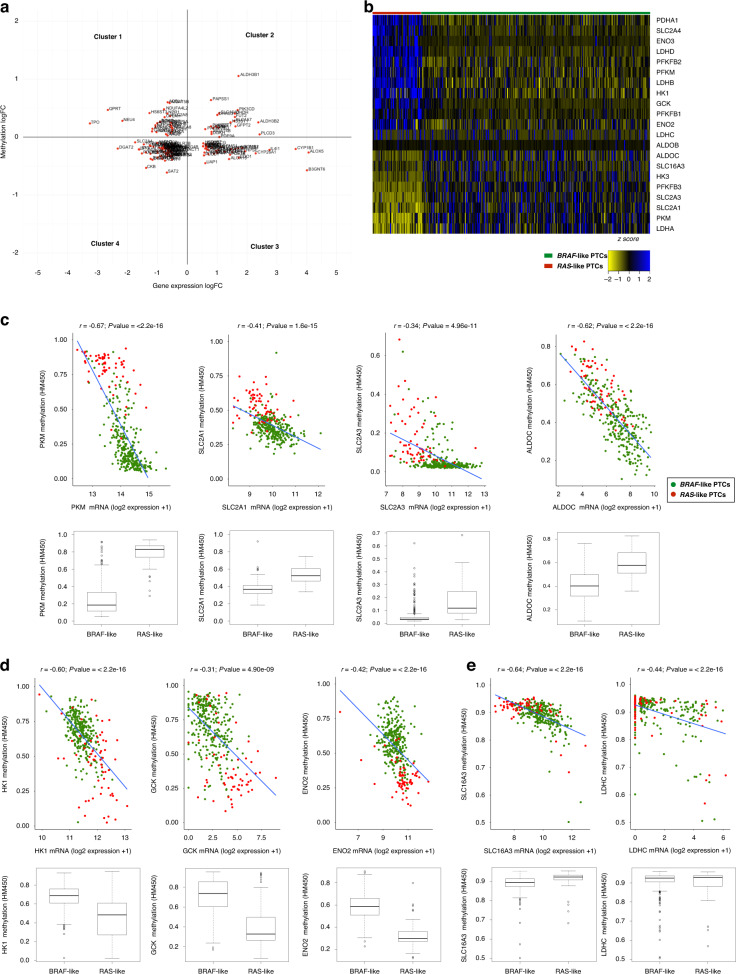
PTC subtypes do not display marked differences in DNA methylation of glucose transporters and glycolytic genes. **a** Scatter plot of expression and methylation data (logFC; THCA datasets) of genes differentially expressed between *BRAF*- and *RAS*-like PTCs. Four distinct clusters of genes have been identified (clockwise from upper left) as altered in *BRAF*-like tumours: hypermethylated and downregulated (cluster 1); hypermethylated and overexpressed (cluster 2); hypomethylated and overexpressed (cluster 3); hypomethylated and downregulated (cluster 4). **b** Heatmap showing normalised expression data (RNA-Seq datasets from THCA cohort; *n* = 398) of genes encoding glucose transporters and glycolysis-related proteins (selected according to KEGG database) in *BRAF*- (green) vs *RAS*-like (red) PTCs. **c**–**e** Scatter plots showing the correlation—by linear regression analysis—between normalised expression (log_2_ scale) and methylation data (logFC; THCA cohort) of 9 selected genes differentially expressed between PTC subtypes. These genes were selected as showing a good (0.30 > *r* < 0.49) or strong (0.5 > *r* < 0.7) correlation (Pearson’s coefficient) and distinguished in significantly hypomethylated and overexpressed (**c**), hypermethylated and downregulated in *BRAF*-like subtype (**d**) and highly correlated, but not differentially methylated, between tumour subtypes (**e**).

To identify the mechanisms underlying the specific metabolic signatures of *BRAF*-like PTCs, we first analysed DNA methylation, the main epigenetic alteration participating in the transcriptional regulation of metabolic genes in cancer [50–52]. Taking advantage of TCGA methylation data available for PTC (THCA dataset), we identified differentially methylated probes (DMPs) in CpG islands. Focusing on CpG islands of all metabolic genes differentially expressed between *BRAF*- and *RAS-*like tumours (i.e., 459 genes) we found that about 30% of them display significant methylation changes (i.e., 144 genes, of which 48 hyper- and 96 hypomethylated; Supplementary Files S1 and S2). Thus, considering the mean methylation values and expression levels of each independent gene, we classified these metabolic genes into four clusters (Fig. 2a), focusing on those belonging to cluster 1 (i.e., 28 genes characterised by CpG islands’ hypomethylation paralleled by overexpression in *BRAF*-like tumours) and cluster 3 (i.e., 37 genes hypermethylated and downregulated in *BRAF*-like tumours; Supplementary File S2).

Interestingly, using KEGG pathway classification, we observed that lipid and nucleic acid metabolism are enriched for differentially methylated genes (Supplementary Fig. S7A and Supplementary File S1). However, CpG islands of genes encoding glucose transporters and glycolysis-related enzymes do not display—except *PFKFB3* (Supplementary Fig. S7A)— significant methylation changes between tumour subtypes. Hence, for these genes, we extended the analysis of methylation from promoters to gene bodies. Moreover, we used gene expression data of each independent tumour sample to measure the correlation index for all these genes. Interestingly, for 9 out of 21 genes differentially expressed between PTC subtypes (Fig. 2b) we measured good (0.30 > *r* < 0.49) or strong (0.5 > *r* < 0.7) correlation (Pearson’s coefficient) between global gene methylation and expression. Using this approach, we could define three different classes of genes: (1) significantly hypomethylated—and overexpressed—in *BRAF*-like tumours (Fig. 2c) and, conversely, (2) hypermethylated—and downregulated—in the same tumour subtype (Fig. 2d), and finally (3) highly correlated but not differentially methylated among tumour subtypes (Fig. 2e). The finding that altered expression of genes responsible for glucose uptake and glycolysis in *BRAF*-like tumours is not supported, at least convincingly, by their differential methylation, makes these tumours possibly not amenable for epigenetic drugs.

**Fig. 3:**
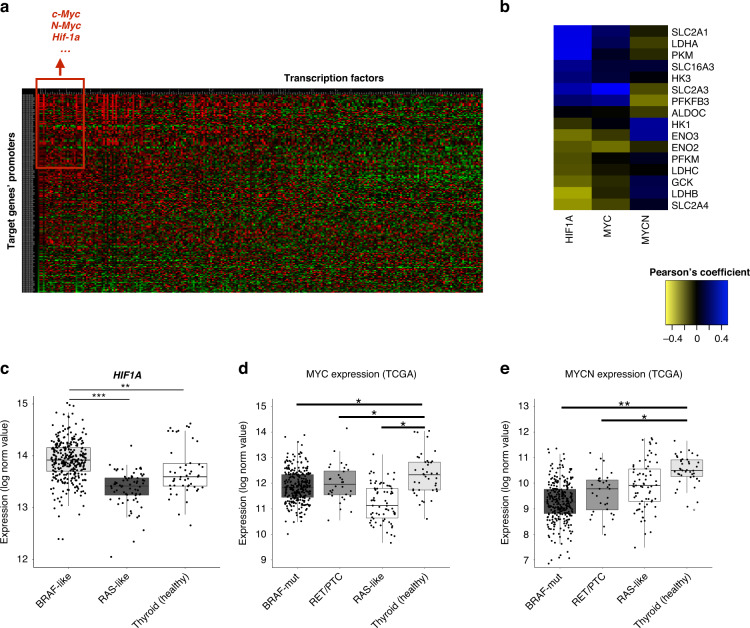
Identification of transcription factors potentially contributing to the metabolic rewiring in *BRAF*-like PTCs. **a** Heatmap showing the enrichment of predicted transcription factors’ binding sites in the putative promoters (window of 1 kb up- and downstream the transcription start site, TSS) of metabolic genes differentially expressed between *BRAF*- and *RAS*-like PTCs. Red squares indicate higher enrichment scores for TFs’ predicted binding. **b** Heatmap showing Pearson’s coefficients between the expression values of top-ranked TFs—having the highest number of predicted binding sites in the promoter of metabolic genes—and the glucose transporters and other glycolytic genes differentially expressed between *BRAF*- and *RAS*-like PTCs (THCA cohort). **c** Box plot comparing normalised *HIF1A* expression values (log_2_ scale) in *BRAF*-, *RAS*-like PTCs and in healthy thyroid samples. ***FDR ≤ 0.001. **d**, **e** Box plots comparing normalised *MYC* (D) and *MYCN* (E) expression values (log_2_ scale) in *BRAF*-mutated, RET/PTC, *RAS*-like PTCs and in healthy thyroid samples (THCA cohort). *FDR ≤ 0.05, **FDR ≤ 0.01.

Thus, we investigated transcription factors (TFs) as the most likely candidates by screening the promoters of metabolism-related DEGs and searching for TFs motifs’ enrichment. Interestingly, c-Myc, n-Myc and Hif-1α were the three top-ranked TFs with the highest number of predicted binding sites (Fig. 3a), in line with the notion that c-Myc overexpression in various cancers [53–55] positively modulates the expression of genes involved in glucose metabolism [56–59]. In addition, Hif-1α and c-Myc crosstalk has been well documented [55, 56], and the glycolytic phenotype of BRAFV600E-mutated melanoma is known to be directly linked to the increase of Hif-1α and c-Myc TFs, which regulate the expression of metabolic genes [9, 60]. Combining TCGA RNA-Seq data and co-expression network data implemented in cBioPortal (https://www.cbioportal.org/), we used a guilt-by-association approach to determine which among top-ranked TFs is most likely to regulate the expression of the glucose transporters and other glycolysis-related genes, which we identified as specifically perturbed in *BRAF*-like tumours. Using this approach, we found that *HIF1A* gene has the highest correlation coefficients (Fig. 3b)—especially with genes reported as glycolysis drivers [35]—and is significantly overexpressed in *BRAF*- (vs *RAS*-like) PTCs (Fig. 3c). Conversely, *MYC* and *MYCN* genes display very low correlation (Fig. 3b) and show only a very modest induction in the *BRAF*- (vs RAS-like) tumour subgroup and are not differentially expressed in tumours vs healthy samples’ comparison (Fig. 3d, e). Overall, these data suggest Hif-1α as a potential regulator of energy metabolism genes specifically altered in *BRAF*-like PTCs.

### Hif-1α modulates the transcription of genes responsible of the glycolytic phenotype in *BRAF*-mutated PTC cells

**Fig. 4:**
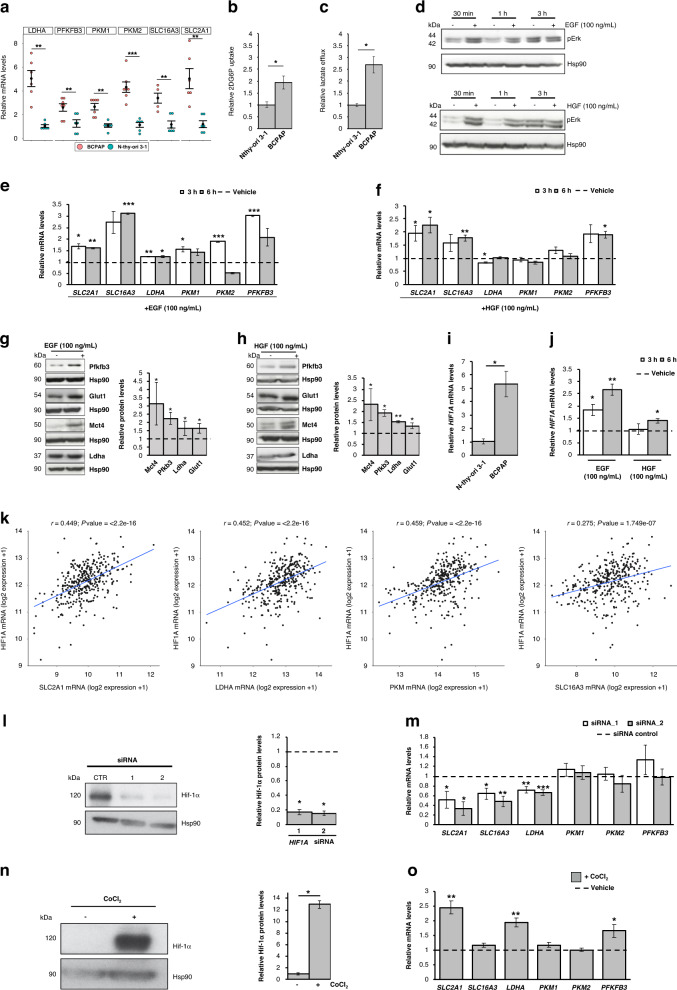
*BRAF*-mutated PTC cells display a glycolytic phenotype mediated by Hif-1α. **a** Relative mRNA quantification (qPCR) of selected metabolic genes in BCPAP compared to normaloid thyroid cell line (Nthy-ori 3-1). Data are reported as mean ± SEM vs Nthy-ori 3-1 cells of at least six independent experiments. *PPIA* was used as reference gene. **P value ≤0.01 and ***P value ≤0.001. **b** Relative colorimetric detection of 2-DG6P uptake in BCPAP compared to Nthy-ori 3-1 cells. Corrected values (pmol) were normalised for the related AUC and data are reported as mean ± SEM of at least four independent experiments. *P value ≤ 0.05. **c** Relative colorimetric detection of l-lactic acid content in the cell culture supernatant of BCPAP compared to Nthy-ori 3-1 cells. Lactate concentration (mmol/L) was normalised for the related AUC and data are reported as mean ± SEM of at least four independent experiments. *P value ≤0.05. **d** Representative autoradiographs of the western blot analysis for pErk in Nthy-ori 3-1 treated or not with Egf (upper panel) or Hgf (lower panel) 100 ng/mL at multiple time points. Hsp90 was used as a loading control. **e**, **f** Relative mRNA quantification (qPCR) of selected metabolic genes in Nthy-ori-3-1 cells upon EGF (100 ng/mL; **e**) and HGF (100 ng/mL; **f**) treatment (3, 6 h). Data are reported as mean ± SEM vs control cells (vehicle-treated; dotted line) of three independent experiments. *PPIA* was used as reference gene. *P value ≤0.05, **P value ≤0.01 and ***P value ≤0.001. **g**, **h** Representative autoradiographs of the western blot analysis for selected metabolic enzymes and transporters (i.e., Pfkfb3, Glut1, Mct4 and Ldha) in Nthy-ori 3-1 treated or not with EGF (**g**) or HGF (**h**) 100 ng/mL at multiple time points. Hsp90 was used as a loading control. Bar graphs (right part of each panel) report relative protein levels normalised on Hsp90 expression (pixel density analysis of western blots). Data are reported as mean ± SEM vs control cells (i.e., treated with the vehicle) of three independent experiments. *P value ≤0.05, **P value ≤0.01. **i**, **j** Relative mRNA quantification (qPCR) of *HIF1A* gene in BCPAP vs Nthy-ori 3-1 (**i**) and in BCPAP treated with EGF and HGF (100 ng/Ml, **j**) for 3 and 6 h vs control cells (i.e., treated with the vehicles; dotted line). Data are reported as mean ± SEM of three independent experiments. *PPIA* was used as a reference. *P value ≤0.05 and **P value ≤0.01. **k** Scatter plot of normalised expression data (log2 scale) showing the correlation—by linear regression analysis—of *HIF1A* and selected glycolysis-related genes in THCA cohort (expression data downloaded from cBioPortal). Pearson correlation coefficient (*r*) and *P* value (*P*) are shown. **l** Representative autoradiographs of western blot analysis (left panel) of Hif-1α protein levels in BCPAP transfected with two different *HIF1A* siRNAs. Hsp90 was used as a loading control. Bar graphs (right panel) report relative Hif-1α levels normalised on Hsp90 expression (pixel density analysis of western blots). Data are reported as mean ± SEM vs control cells (i.e., BCPAP transfected with scrambled siRNAs; dotted line) of three independent experiments. *P value ≤0.05. **m** Relative mRNA quantification (qPCR) of selected metabolic genes upon *HIF1A* silencing in BCPAP. Data are reported as mean ± SEM vs control cells (scrambled siRNAs; dotted line) of at least three independent experiments. *PPIA* was used as reference. *P value ≤0.05, **P value ≤0.01 and ***P value ≤0.001. **n** Representative autoradiographs of western blot analysis (left panel) of Hif-1α protein levels in BCPAP treated with CoCl_2_ (250 µM, 24 h). Hsp90 was used as a loading control. Bar graphs (right panel) report relative Hif-1α levels normalised on Hsp90 expression (pixel density analysis of western blots). Data are reported as mean ± SEM vs control cells (i.e., treated with the vehicle) of three independent experiments. *P value ≤0.05. **o** Relative mRNA quantification (qPCR) of selected metabolic genes in BCPAP treated with CoCl_2_ (250 µM, 24 h). Data are reported as mean ± SEM vs control cells (BCPAP treated with the vehicle; dotted line) of at least four independent experiments. *PPIA* was used as a reference. *P value ≤0.05 and **P value ≤0.01.

The analysis of transcriptome data from TCGA overall indicates a glycolytic phenotype in *BRAF*-like PTCs. Thus, to experimentally validate this metabolic gene signature, we compared a BRAFV600E-mutated papillary (BCPAP) tumour cell line and the *BRAF* wild-type normaloid thyroid follicular epithelial cell line (Nthy-ori 3-1). For the expression analysis, we selected glycolysis-related genes (i.e., *SLC2A1*, *SLC16A3*, *PFKFB3*, *PKM1*, *PKM2* and *LDHA*) (i) being the most overexpressed in *BRAF*- vs *RAS*-like tumours, (ii) displaying the highest glycolytic flux’s coefficients according to Tanner and colleagues [49] and (iii) being also unfavourable prognostic markers across multiple tumour types according to The Human Protein Atlas database (https://www.proteinatlas.org/). Notably, BCPAP cells display the overexpression of selected genes (Fig. 4a) and increased glucose uptake (Fig. 4b) and lactate efflux (Fig. 4c) compared to Nthy-ori 3-1 cells, confirming the high glycolytic phenotype of *BRAF*-mutated tumours. Afterwards, we evaluated if MAPK activation alone—in non-tumoral thyroid cells—is sufficient to transcriptionally induce the glycolytic genes. Interestingly, most of them are overexpressed upon short-term treatment with two distinct MAPK activators, ie., epidermal and hepatocyte growth factors (EGF and HGF, respectively, Fig. 4d–h). Moreover, in line with the finding that *HIF1A* is overexpressed in *BRAF*-like tumour samples, *BRAF*-mutated cells display increased levels of *HIF1A* compared to Nthy-ori 3-1 cells (Fig. 4i). Moreover, this TF is also induced by EGF and HGF in normaloid cells (Fig. 4j). Interestingly, we found that all (except *PFKFB3*) selected metabolic genes—upregulated in BCPAP cells and induced by MAPK stimulation (Fig. 4a, e–h)—display robust co-expression with *HIF1A* in the THCA cohort (Fig. 4k). Thus, to understand if Hif-1α regulates the expression of these metabolic genes, we used two distinct siRNAs targeting *HIF1A* mRNA to knock it down in *BRAF*-mutated tumour cells (Fig. 4l and Supplementary Fig. S7B). Interestingly, as reported in Fig. 4m, tumour cells depleted for Hif-1α display a significantly reduced expression of *SLC2A1*, *LDHA* and *SLC16A3*. Moreover, tumour cells cultured in the presence of CoCl_2_—a known hypoxia-mimicking chemical inducing Hif-1α protein stabilisation (Fig. 4n and Supplementary Fig. S7C)—display a marked induction of *SLC2A1*, *LDHA* and *PFKFB3* (Fig. 4o), further indicating that this transcription factor contributes to positively modulate glycolysis-related genes altered in *BRAF*-like PTCs.

### Hif-1α-mediated glycolytic phenotype of *BRAF*-mutated PTC cells is attenuated by BRAFi

**Fig. 5:**
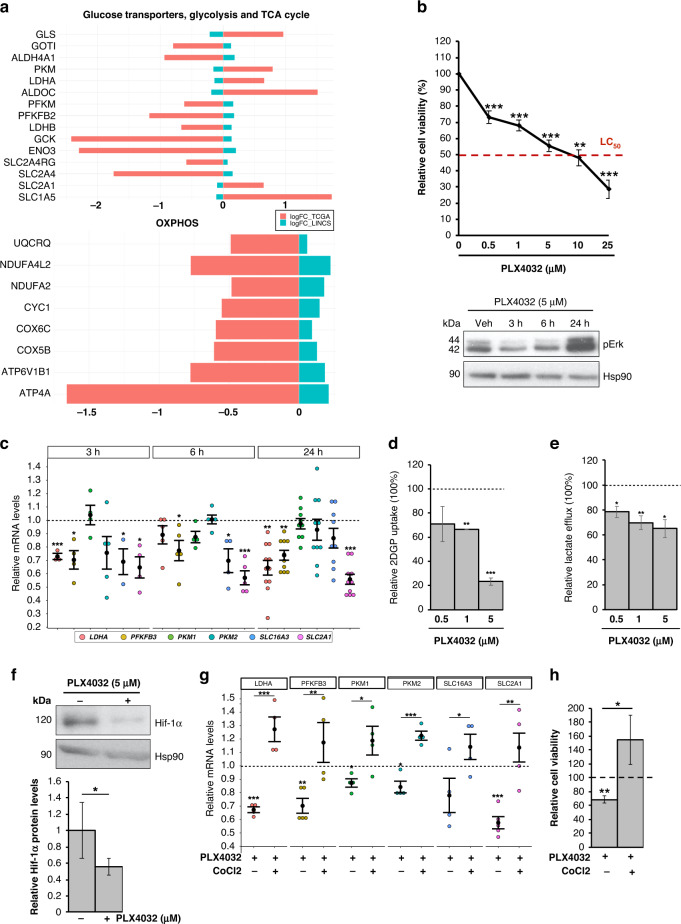
BRAFi counteract the glycolytic phenotype and Hif-1α-modulated transcription signature of *BRAF*-mutant PTC cells. **a** Bar graphs indicate the expression levels (logFC) of genes involved in energy metabolism—i.e., glucose transport, glycolysis, TCA cycle (upper panel) and OXPHOS (lower panel)—and differentially expressed between *BRAF*- and *RAS*-like tumours, whose expression is reverted upon treatment with vemurafenib (blue bars) in multiple *BRAF*-mutated tumour cell lines from the LINCS 1000 Project. TCGA data (THCA cohort) for the same genes are indicated as red bars (logFC). **b** Relative cell viability (percentage; upper panel) in BCPAP cells upon treatment with PLX4032 treatment (0.5, 1, 5, 10 and 25 µM)—a vemurafenib analogue—for 72 h. Red dotted line indicates the median lethal concentration (lethal concentration 50%, LC_50_). Data are reported as mean ± SEM vs control cells (i.e., BCPAP treated with Veh; set to 100% of viability) of at least four independent experiments. **P value ≤0.01 and ***P value ≤0.001. Representative autoradiographs (lower panel) of western blot analysis of Erk phosphorylation (i.e., pErk) levels in BCPAP treated with PLX4032 (5 µM; 3, 6 and 24 h). Hsp90 was used as a loading control. **c** Relative mRNA quantification (qPCR) of selected metabolic genes in BCPAP treated with PLX4032 (5 µM; 3, 6 and 24 h). Data are reported as mean ± SEM vs control cells (i.e., treated with the vehicle; dotted line) of at least four independent experiments. *P value ≤0.05, **P value ≤0.01 and ***P value ≤0.001. **d** Relative colorimetric detection of total 2-DG6P uptake in BCPAP treated with different concentrations of PLX4032 for 72 h. Corrected values (pmol) were normalised for the related AUC and data are reported as mean ± SEM of at least three independent experiments. The effect of each treatment was estimated as the percentage of glucose uptake of control cells (i.e., BCPAP treated with the vehicle, set to 100%; dotted line). **P value ≤0.01 and ***P value ≤0.01. **e** Relative colorimetric detection of l-lactic acid content in cell culture supernatant of BCPAP treated with different concentrations of PLX4032 for 72 h. Lactate concentration (mmol/L) was normalised for the related AUC and data are reported as mean ± SEM of at least four independent experiments. The effect of each treatment was estimated as the percentage of lactate secreted by control cells (i.e., BCPAP treated with the vehicle, set to 100%; dotted line). *P value ≤0.05 and **P value ≤0.01. **f** Representative autoradiographs of western blot analysis (upper panel) of Hif-1α protein levels in BCPAP treated with PLX4032 (5 µM; 24 h). Hsp90 was used as a loading control. Bar graphs (lower panel) report relative Hif-1α levels normalised on Hsp90 expression (pixel density analysis of western blots). Data are reported as mean ± SEM vs control cells (i.e., BCPAP treated with the vehicle) of three independent experiments. *P value ≤ 0.05. **g** Relative mRNA quantification (qPCR) of selected metabolic genes in BCPAP upon PLX4032 treatment (5 µM) alone—vs cells treated with the vehicle—or in combination with CoCl_2_ (125 µM)—vs cells treated with CoCl_2_ alone—for 24 h. Data are reported as mean ± SEM vs control cells (dotted line) of at least four independent experiments. *PPIA* was used as reference. *P value ≤0.05, **P value ≤0.01 and ***P value ≤0.001. **h** Relative cell viability in BCPAP upon PLX4032 treatment (5 µM) alone—vs cells treated with the vehicle—or in combination with CoCl_2_ (125 µM)—vs cells treated with CoCl_2_ alone—for 72 h. Data are reported as mean ± SEM vs control cells (dotted line, set to 100% of viability) of four independent experiments. *P value ≤0.05 and **P value ≤0.01.

The inhibition of oncogenic B-raf by VMR has been shown to impair glucose uptake and glycolysis in *BRAF*-mutated melanoma [9]. In the same tumour type, BRAFV600E-mediated regulation of glycolysis has been demonstrated to occur at the transcriptional level [9]. Hence, to address if the VMR-mediated transcriptional effect on glycolysis-related genes is common to other *BRAF*-mutated tumours, we analysed public RNA-Seq datasets from LINCS L1000 Project. Interestingly, we disclosed that VMR treatment has a marked transcriptional effect—for several metabolic genes—that is shared across multiple *BRAF*-mutated tumour cell lines. Indeed, as reported in Fig. 5a, VMR reverts the expression of a large fraction of genes involved in glucose uptake, glycolysis, TCA cycle and OXPHOS, identified as hallmarks of *BRAF*-like tumours (Supplementary File S1). This analysis, other than corroborating the anti-glycolytic effect of VMR also in other *BRAF*-mutated tumour types, uncovered also a previously unrecognised repressive effect on genes belonging to lipid (Supplementary Fig. S7D), nucleotide (Supplementary Fig. S7E, F) and amino acid metabolism pathways (Supplementary Fig. S7G).

To experimentally verify the transcriptional effect of VMR on metabolic genes in the context of thyroid carcinomas, we evaluated if PLX4032—a VMR analogue—could repress the expression of glycolysis-related genes in a *BRAF*-mutated PTC cell line. We first evaluated BCPAP cells’ viability at increasing drug doses (Fig. 5b, upper panel). Notably, we assessed that PLX4032 concentrations lower than LC_50_ are sufficient to inhibit ERK phosphorylation even at early time points (3, 6 h; Fig. 5b, lower panel). However, in line with the report from Montero-Conde et al. [61], MAPK pathway inhibition by PLX4032 induces a rapid rebound of pErk (24 h; Fig. 5b, lower panel). As we measured a marked reduction of tumour cells viability 72 h upon PLX4032 treatment we explored the possibility that this cytotoxic effect may be due to metabolic restraining. Interestingly, a transcriptional repression of the selected metabolic genes (Fig. 5c) and a reduction of glucose uptake (Fig. 5d and Supplementary Fig. S8A) and lactate efflux (Fig. 5e) was observed in *BRAF*-mutated PTC cells. Notably, in line with our finding that Hif-1α contributes to the glycolytic phenotype of *BRAF*-mutated cells, PLX4032 treatment also impaired Hif-1α levels (Fig. 5f). Accordingly, Hif-1α stabilisation is able to rescue both the expression of glycolysis-related genes (Fig. 5g) and tumour cells’ viability (Fig. 5h) induced by B-raf inhibition. These findings indicate that the attenuation of the glycolytic phenotype induced by B-raf inhibition is mediated—at least in part—by the transcription factor Hif-1α, which controls the expression of a network of genes involved in energy metabolism and specifically altered in *BRAF*-like tumour samples.

### The antitumoral effect of BRAFi on *BRAF*-mutated PTC and ATC cells is potentiated by the combination with diclofenac

**Fig. 6:**
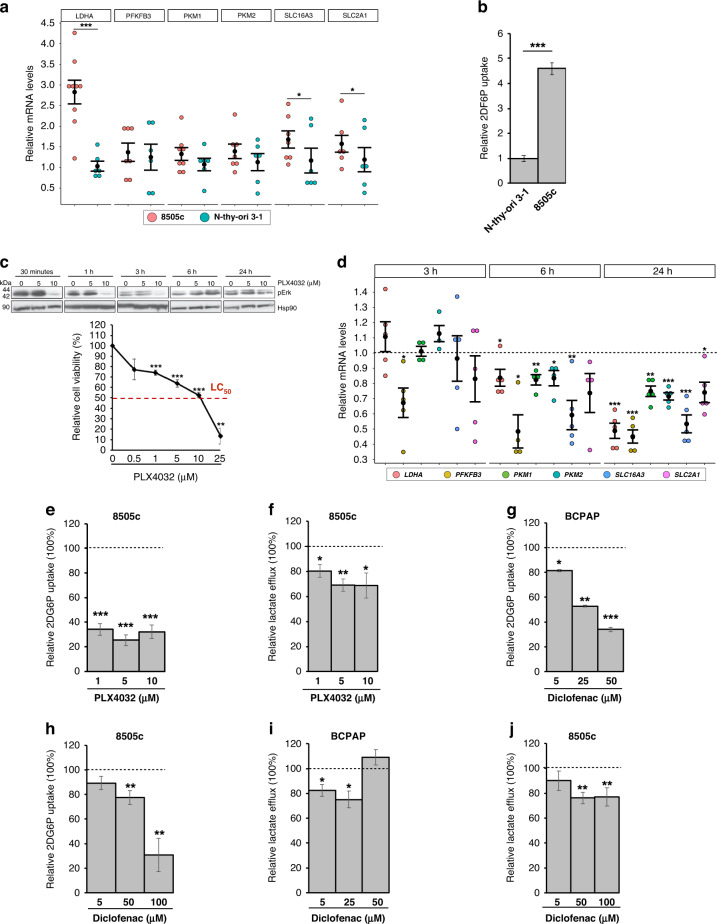
*BRAF*-mutated PTC and ATC cells display a similar glycolytic phenotype modulated by BRAFi and diclofenac. **a** Relative mRNA quantification (qPCR) of selected metabolic genes in 8505c compared to Nthy-ori 3-1. Data are reported as mean ± SEM vs Nthy-ori 3-1 cells of at least six independent experiments. *PPIA* was used as reference. *P value ≤0.05 and ***P value ≤0.001. **b** Relative colorimetric detection of total 2-DG6P uptake in 8505c compared to Nthy-ori 3-1 cells. Corrected values (pmol) were normalised for the related AUC and data are reported as mean ± SEM of at least four independent experiments. ***P value ≤0.01. **c** Representative autoradiographs of western blot analysis (upper panel) of Erk phosphorylation (i.e., pErk) levels in 8505c cells treated with PLX4032 (5 µM and 10 µM; 30 min, 1, 3, 6 and 24 h). Hsp90 was used as a loading control. Relative cell viability (percentage; lower panel) in 8505c cells upon treatment with PLX4032 treatment (0.5, 1, 5, 10 and 25 µM)—a vemurafenib analogue—for 72 h. Red dotted line indicates the median lethal concentration (lethal concentration 50%, LC_50_). Data are reported as mean ± SEM vs control cells (i.e., BCPAP treated with the vehicle; set to 100% of viability) of at least four independent experiments. **P value ≤0.01, ***P value ≤0.001. **d** Relative mRNA quantification (qPCR) of selected metabolic genes in 8505c treated with PLX4032 (10 µM; 3, 6 and 24 h). Data are reported as mean ± SEM vs control cells (i.e., 8505c treated with the vehicle; dotted line) of at least four independent experiments. *P value ≤0.05, **P value ≤0.01 and ***P value ≤0.001. **e** Relative colorimetric detection of total 2-DG6P uptake in 8505c treated with different concentrations of PLX4032 for 72 h. Corrected values (pmol) were normalised for the related AUC and data are reported as mean ± SEM of at least four independent experiments. The effect of each treatment was estimated as the percentage of glucose uptake of control cells (i.e., 8505c treated with the vehicle, set to 100%; dotted line). **P value ≤0.01 and ***P value ≤0.01. **f** Relative colorimetric detection of l-lactic acid content in cell culture supernatant of 8505c treated with different concentrations of PLX4032 for 72 h. Lactate concentration (mmol/L) was normalised for the related AUC and data are reported as mean ± SEM of at least four independent experiments. The effect of each treatment was estimated as the percentage of lactate secreted by control cells (i.e., 8505c treated with the vehicle, set to 100%; dotted line). *P value ≤0.05 and **P value ≤0.01. **g**, **h** Relative colorimetric detection of total 2-DG6P uptake in BCPAP (**g**) and 8505c (**h**) treated with different concentrations of diclofenac for 72 h. Corrected values (pmol) were normalised for the related AUC and data are reported as mean ± SEM of at least three independent experiments. The effect of each treatment was estimated as the percentage of glucose uptake of control cells (i.e., cells treated with the vehicle, set to 100%; dotted line). *P value ≤0.05, **P value ≤0.01 and ***P value ≤ 0.01. **i**, **j** Relative colorimetric detection of l-lactic acid content in cell culture supernatant of BCPAP (**i**) and 8505c (**j**) treated with different concentrations of diclofenac for 72 h. Lactate concentration (mmol/L) was normalised for the related AUC and data are reported as mean ± SEM of at least four independent experiments. The effect of each treatment was estimated as the percentage of lactate secreted by control cells (i.e., cells treated with the vehicle, set to 100%; dotted line). *P value ≤0.05 and **P value ≤0.01.

**Fig. 7:**
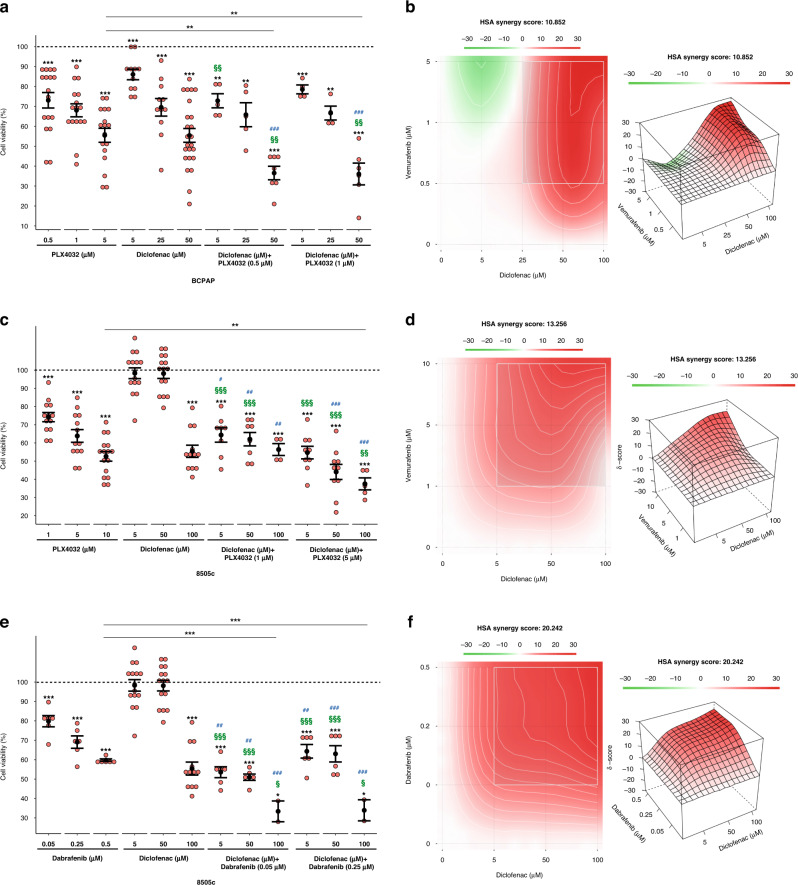
The combination of BRAFi and diclofenac synergistically reduces the cell viability of *BRAF*-mutated PTC and ATC cells. **a** Relative cell viability in BCPAP cells upon single (PLX4032 and diclofenac) and combined treatment with the two drugs at different doses for 72 h. Data are reported as mean ± SEM vs control cells (i.e., BCPAP treated with the vehicles—indicated as dotted line—are set to 100% of viability) of at least four independent experiments. **P value ≤0.01 and ***P value ≤0.001 vs control cells or BCPAP treated with the maximum dose of PLX4032 (5 µM). ^###^*P* value ≤0.001 vs BCPAP treated with the same doses of PLX4032 alone. ^§§^*P* value ≤ 0.01 vs BCPAP treated with the same doses of diclofenac alone. **b** Heatmap showing—in 2D (left panel) and 3D (right panel)—the HSA synergy scores (i.e., positive values in red denote synergy), analysed by SynergyFinder tool, for the combination of PLX4032 and diclofenac in BCPAP. **c** Relative cell viability in 8505c cells upon single (PLX4032 and diclofenac) and combined treatment with the two drugs at different doses for 72 h. Data are reported as mean ± SEM vs control cells (i.e., 8505c treated with the vehicles—indicated as dotted line—are set to 100% of viability) of at least four independent experiments. ***P value ≤0.001 vs control cells or 8505c treated with the maximum dose of PLX4032 (10 µM). ^#^*P* value ≤0.05, ^##^*P* value ≤0.01 and ^###^*P* value ≤0.001 vs 8505c treated with the same doses of PLX4032 alone. ^§§^*P* value ≤0.01 and ^§§§^*P* value ≤0.01 *vs* 8505c treated with the same doses of diclofenac alone. **d** Heatmap showing—in 2D (left panel) and 3D (right panel)—the HSA synergy scores (i.e., positive values in red denote synergy), analysed by SynergyFinder tool, for the combination of PLX4032 and diclofenac in 8505c. **e** Relative cell viability in 8505c cells upon single (dabrafenib or diclofenac) and combined treatment with the two drugs at different doses for 72 h. Data are reported as mean ± SEM vs control cells (i.e., 8505c treated with the vehicles—indicated as dotted line—are set to 100% of viability) of at least three independent experiments. *P value ≤0.05 and ***P value ≤0.001 vs control cells or 8505c treated with the maximum dose of DBR (0.5 µM). ^##^*P* value ≤0.01 and ^###^*P* value ≤0.001 vs 8505c treated with the same doses of dabrafenib alone. ^§^*P* value ≤0.05 and ^§§§^*P* value ≤0.01 vs 8505c treated with the same doses of diclofenac alone. **f** Heatmap showing—in 2D (left panel) and 3D (right panel)—the HSA synergy scores (i.e., positive values in red denote synergy), analysed by SynergyFinder tool, for the combination of dabrafenib and diclofenac in 8505c.

As 20–25% of the aggressive ATC cases display BRAFV600E mutation, we sought to verify, likewise reported in *BRAF*-driven PTCs, the presence of a *BRAF*-mediated glycolytic phenotype in this tumor and if B-raf inhibitors are able to restrain it. Notably, the highly aggressive anaplastic *BRAF*-mutated tumour cells (i.e., 8505c)—compared to normal thyroid cells—display higher levels of multiple metabolic genes (Fig. 6a) and increased glucose uptake (Fig. 6b). Moreover, in line with the notion that B-raf inhibition by vemurafenib is effective on BRAFV600E-mutated ATCs [62–65], PLX4032 markedly reduces pErk and cell viability of ATC cells (Fig. 6c), likewise the treatment with trametinib, an FDA-approved MEK inhibitor (MEKi) for ATC (Supplementary Fig. S8B). Noteworthy, we found that it impairs the glycolytic phenotype also of these aggressive tumour cells, by transcriptionally repressing glycolysis-related genes (Fig. 6d) and reducing glucose uptake (Fig. 6e and Supplementary Fig. S8A) and lactate efflux (Fig. 6f). Further validating the MAPK-dependent modulation of the metabolic phenotype and of glycolytic genes, similar results were obtained using trametinib (Supplementary Fig. S8C, D). Considering that the combination of BRAFi and MEKi is currently used in the therapy of *BRAF*-mutated ATC, 8505c cells were treated with PLX4032 and trametinib combination. Interestingly, despite the marked suppression of pErk (Supplementary Fig. S8E), tumour cell viability was not significantly impaired by BRAFi and MEKi combination compared to the single drug treatments (Supplementary Fig. S8F). Accordingly, no synergistic effect has been disclosed for PLX4032 and trametinib combination (Supplementary Fig. S8G), further indicating the need to identify other drugs able to maximise the BRAFi effects. Thus, we sought to exploit metabolic pathways as possible targets for new promising anti-cancer strategies. Indeed, several trials based on the simultaneous targeting of multiple pathways are currently evaluating the effects of combined therapies in *BRAF*-mutated tumours, including in PTC and especially ATC. In this context, non-steroidal anti-inflammatory drugs (NSAIDs)—commonly used as COX inhibitors—have been proposed as VMR-sensitisers in *BRAF*-mutated melanoma [10]. Hence, we exploited the glycolytic dependency of *BRAF*-driven thyroid carcinomas using combinations of NSAIDs and BRAFi. To this aim, we selected diclofenac - reported to restrain glycolytic flux in melanoma [10, 31, 66]—as a candidate drug. Notably, diclofenac has been recently reported as a possible inhibitor of Glut1, Mct4 and Ldha [67]. Hence, we treated *BRAF*-mutated PTC and ATC cells with different doses of diclofenac. We found that such a treatment has no effect on MAPK signalling (Supplementary Fig. S9A), as well as on the expression of both *HIF1A* and glycolysis-related genes at multiple time points in both tumour cell lines (Supplementary Fig. S9B–F). However, more interestingly, diclofenac is able to impair glucose uptake (Fig. 6g, h, in PTC and ATC, respectively, and Supplementary Fig. S9G) and lactate excretion (Fig. 6i, j, in PTC and ATC, respectively) in both the *BRAF*-mutated tumour cell lines. Thus, the effect of diclofenac on the energy metabolism is not due to transcriptional repression—as it occurs for B-raf inhibition by PLX4032—but rather it may depend on the inhibition of key glycolytic enzymes. In line with the ability of these drugs to target distinct pathways/processes, we assessed that diclofenac acts synergistically with PLX4032 in reducing cell viability in both tumour types (Fig. 7a–d). We further confirmed the synergistic effect of NSAID and BRAFi combining diclofenac and dabrafenib (DBR), a B-raf inhibitor currently approved by FDA in combination with trametinib for *BRAF*-mutated ATCs (Fig. 7e, f for ATC and Supplementary Fig. S9H for PTC), further confirming the synergistic effect of NSAID and BRAFi. Taken together, our results suggest that diclofenac potentiates the cytotoxic effect of BRAFi, possibly by inhibiting tumour glycolytic phenotype. Notably, the combination of diclofenac with low doses of BRAFi allows obtaining the same (or more pronounced) effect on cell viability reached by high doses of PLX4032. For instance, in BCPAP cells the co-treatment with diclofenac 50 µM causes a stronger reduction of cell viability even with five to tenfold lower doses of BRAFi (Fig. 7a). Likewise, in 8505c cells the combination of diclofenac 100 µM induces a more marked impairment of cell viability even with two to tenfold lower doses of PLX4032 and DBR (Fig. 7c, e). Thus, the finding that BRAFi/diclofenac combinations exert a potent anti-tumour activity even at very low BRAFi doses in both PTC and ATC *BRAF*-mutated cell lines (Fig. 7a–f) strongly suggests the possibility to delay—or reduce—the onset of acquired resistance, limiting the risk of unwanted effects.

## Discussion

Metabolic reprogramming of tumour cells is among the ten hallmarks of cancer [68] and has been proposed as one of the driver mechanisms able to increase the metastatic potential and reduce drug sensitivity, leading to poor prognosis in multiple cancer types [3–11, 43, 69, 70]. Hence, targeting metabolism is emerging as a promising strategy to improve current therapies and overcome drug resistance. In line with independent studies reporting the association of metabolic gene signatures and clinicopathological features in papillary thyroid carcinomas [20–27], our work provides evidence of a strong perturbation of metabolic genes between the two distinct tumour subtypes (i.e., *BRAF*- and *RAS*-like PTCs). Our results indicate that these tumour subtypes largely differ for the expression of key genes encoding glucose, lactate and glutamine transporters, glycolytic enzymes as well as genes involved in the metabolism of lipids, amino acids and nucleic acids. Particularly, the finding that the altered expression of energy metabolism genes does not associate with a differential methylation pattern makes this metabolic route not amenable for epigenetic drug treatment, at least in the context of PTCs. However, the identification of Hif-1α—overexpressed in *BRAF*-like tumours—as one of the orchestrators of metabolic processes in these tumours, likewise in melanoma [9], opens new intriguing therapeutic perspectives. Indeed, together with c-Myc, Hif-1α is known to simultaneously induce glycolytic genes and inhibitors of mitochondrial metabolism [60, 71–73], to reduce mitochondrial biogenesis [74, 75] and to play a key role in metabolic reprogramming in many tumour types [69, 72, 74–76]. Interestingly, the modulation of Hif-1α expression can affect the sensitivity to radiotherapy in thyroid neoplasia and to chemotherapy in multiple tumours [77], making it a promising therapeutic target for many cancer types, including thyroid carcinomas [78].

**Fig. 8:**
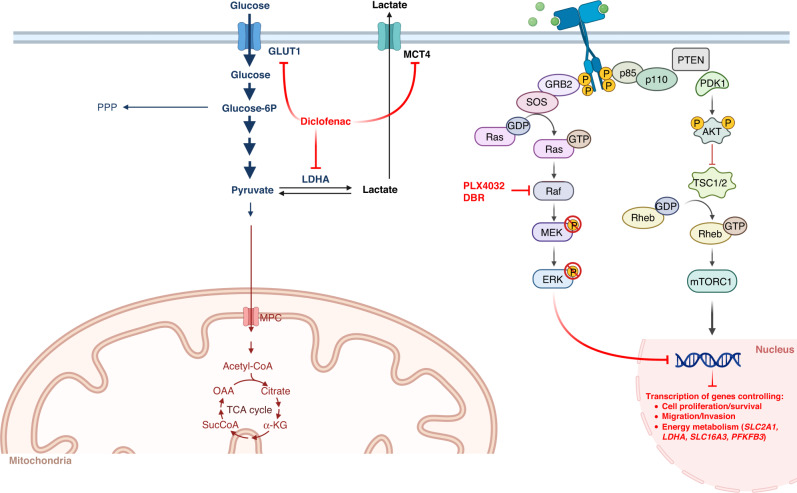
Schematic model of BRAFi and diclofenac effects on metabolic processes. Schematic representation of the possible molecular mechanism underlying the synergism between diclofenac and vemurafenib/dabrafenib (PLX4032 and DBR) in *BRAF*-mutated papillary and anaplastic thyroid carcinomas. Created with Biorender.com.

Overall, our findings indicate the possibility to interfere with energy metabolism and—in the spirit of precision oncology—to specifically target tumour cells. Noteworthy, the identification of subtype-specific signatures of metabolic genes reinforces the need to stratify thyroid carcinoma patients based on their background of somatic mutations. This approach is particularly relevant as *BRAF*-mutated PTCs and ATCs can be selectively targeted by BRAFi—i.e., vemurafenib and dabrafenib (combined with trametinib in ATC)—whose efficacy has been proven both in BRAFV600E-positive metastatic melanoma [79] and in non-melanoma cancers [65, 80]. However, despite sharing the same—and most frequent—*BRAF* mutation (i.e., BRAFV600E), different tumour types display a heterogeneous responsiveness to BRAFi-based therapies. An interesting hypothesis to explain such heterogeneity is that drug effectiveness may depend on a different metabolic vulnerability, reported as the Achilles’ heel of *BRAF*-driven melanoma [8–10]. In line with this, both in PTC and ATC, (i) the overexpression of key glucose transporters and glycolytic enzymes (identified herein as hallmark of papillary and anaplastic *BRAF*-mutated tumours), (ii) their transcriptional repression and (iii) the impairment of glucose uptake and glycolytic flux upon inhibition of MAPK pathway (by targeting B-raf and/or MEK1/2 proteins), indicate that thyroid carcinomas behave similarly to melanoma cells. Although we did not evaluate if impairing the glycolytic flux induces a switch toward OXPHOS in *BRAF*-mutated tumour cells, our results encouraged us to exploit energy metabolism as an additional process to target together with the MAPK pathway. Drug combinations to simultaneously target multiple pathways/processes are under active evaluation in several *BRAF*-mutated tumours, and especially in the most aggressive forms, such as anaplastic tumours. In particular, NSAIDs have been recently proposed as BRAFi-sensitisers in melanoma [10, 31]. Our work indicates that papillary and anaplastic thyroid carcinomas can be included in the growing list of tumours amenable to treatment with combinatorial approaches targeting both canonical pathways (MAPK) and new metabolic ones. Indeed, we report that diclofenac—a well-studied and clinically safe and approved drug—restrains the glycolytic phenotype of *BRAF*-mutated papillary and anaplastic thyroid carcinoma cells, opening new relevant clinical perspectives for these tumours. Moreover, the finding that diclofenac treatment has no effect on the transcriptional modulation of either *HIF1A* or glycolytic genes suggests that its activity on glucose uptake and glycolytic flux is independent from MAPK pathway (Fig. 8). The synergistic effect of diclofenac and PLX4032 on tumour cells viability can be explained by the simultaneous repression of the metabolism-shaping TF Hif-1α (and its metabolic network) and by the inhibition of Ldha enzyme, Glut1 and Mct4 transporters (schematized in Fig. 8). Noteworthy, the repressive effect of diclofenac on tumour metabolism is evident even at low concentrations (25 μM and 50 μM for PTC and ATC cells, respectively), which are below the physiologically relevant plasmatic levels (0.15–105 mg/l, corresponding to ∼50–350 μM) [10, 81, 82] achieved at the recommended doses to treat inflammatory states [83]. Moreover, several independent reports about intravenous administration of diclofenac, showing twofold higher plasma concentrations (vs oral administration) [81, 84–86] and 3-times higher steady-state levels in synovial fluid, suggest that similar drug concentrations may be eventually reached in tumours [10]. More interestingly, diclofenac treatment becomes synergic with PLX4032 and DBR even at very low doses of BRAFi (i.e., 0.5 μM for PTC and 1 μM for ATC), which show only a modest impact on tumour cells viability (20–25% decrease). Noteworthy, our results clearly show that the combination with diclofenac outperforms even the first-line treatment approved for *BRAF*-mutated ATC, i.e., BRAFi plus MEKi (trametinib) combination. This finding acquires higher relevance in light of the urgent need to optimise therapeutic regimens for improving drug sensitivity and overcoming resistance onset and tumour relapse. In this regard, the identification of new pharmacological targets, as well as different drug combinations, appears promising to evaluate new therapeutic strategies. For instance, Ldha inhibition has been recently proposed as an attractive strategy for novel approaches in oncology, especially for targeting tumours highly addicted to aerobic glycolysis (reviewed in [87, 88]). However, because of tumour heterogeneity, Ldha inhibition may be more effective—in some cancers—if combined with other drugs targeting also OXPHOS-addicted cells [88]. Therefore, it would be relevant to test new drug combinations (BRAFi and LDHAi) in *BRAF*-mutated tumours, including—but not limiting to—thyroid carcinomas. Moreover, considering our results and the potential of NSAIDs to target multiple glycolysis-related proteins, it would be interesting to test these drugs in tumours where Ldha inhibition has proven successful in preclinical studies, such as pancreatic and lung cancers. Until now, few studies in glioma [89] and melanoma [10, 31] have evaluated the metabolic effect of the combinatorial use of diclofenac in cancer therapies, whereas most of the studies have focused on its anti-angiogenic, immunomodulatory and pro-apoptotic properties (reviewed in ref. [90]). In conclusion, our results suggest that the combinatorial use of BRAFi and diclofenac is likely to represent a new possible therapeutic approach to treat *BRAF*-mutated papillary and aggressive anaplastic thyroid carcinomas. We envision that diclofenac, having an established role in oncological practice for the treatment of cancer-related pain, may eventually be considered as an active component of new therapies targeting tumour metabolism in *BRAF*-mutated cancers.

## Supplementary information

Supplementary Figures, legends and Table

Supplementary File 1

Supplementary File 2

Supplementary Table

## Data Availability

All data generated or analysed during this study are included either in this article or in additional files, and the links to public datasets are reported in the manuscript.
