# Supplementary material for: Targeting metabolism by B-raf inhibitors and diclofenac restrains the viability of BRAF-mutated thyroid carcinomas with Hif-1α-mediated glycolytic phenotype
Source: Br J Cancer. 2023 May 17;129(2):249–65. doi: 10.1038/s41416-023-02282-2 (PMC10338540; doi:10.1038/s41416-023-02282-2)
Supplement: Supplementary file 1 — Supplementary Figures, legends and Table [file 41416_2023_2282_MOESM1_ESM.pdf]

Supplementary Figures, Figure Legends and title/legends for Supplementary files

A

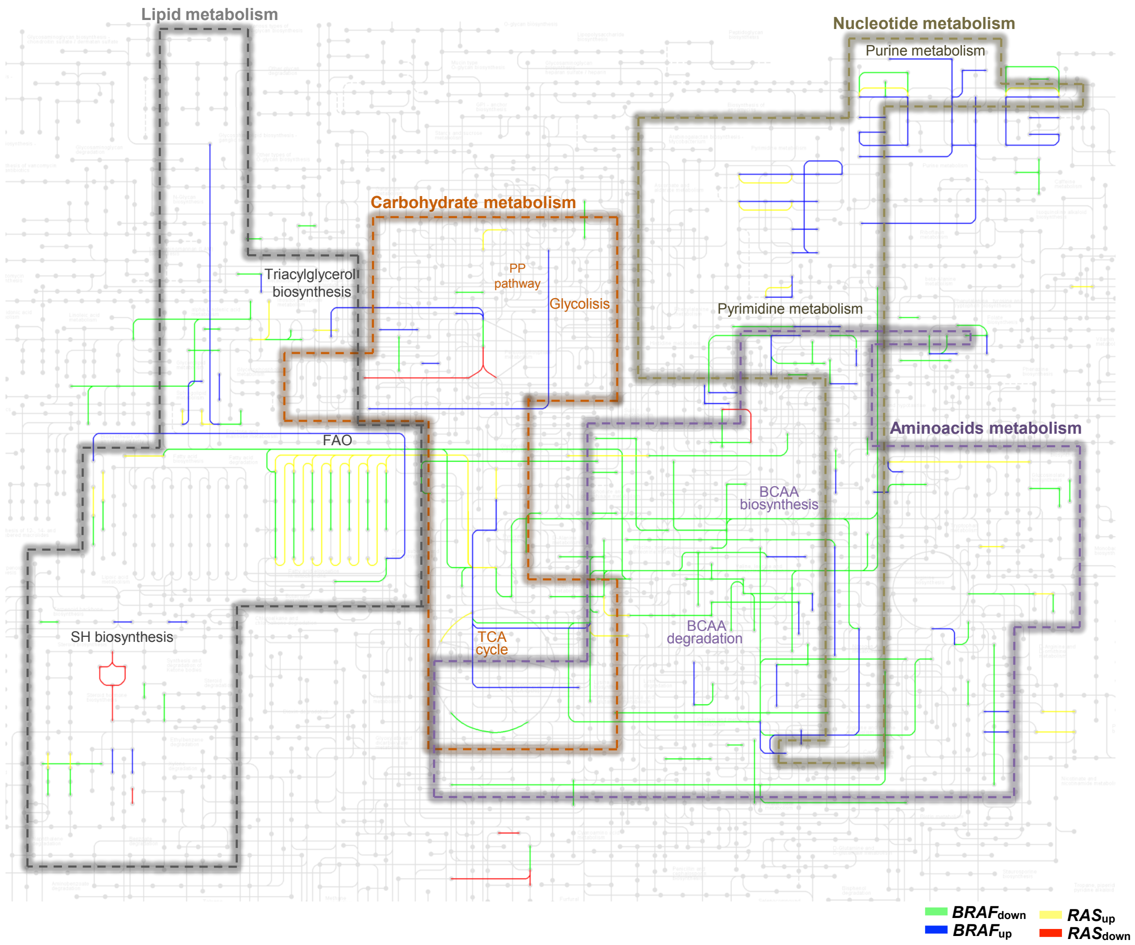

Lipid metabolism
Nucleotide metabolism
Purine metabolism
Carbohydrate metabolism
PP pathway
Glycolisis
Triacylglycerol biosynthesis
Pyrimidine metabolism
FAO
Aminoacids metabolism
BCAA biosynthesis
BCAA degradation
TCA cycle
SH biosynthesis
BRAFdown
BRAFup
RASup
RASdown

B

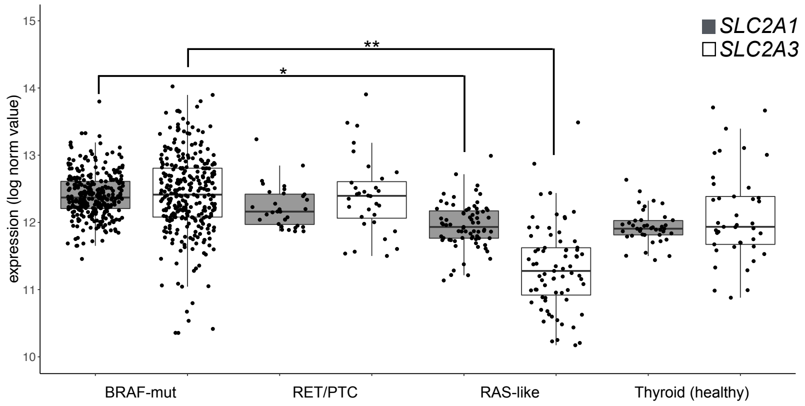

SLC2A1
SLC2A3
**
*
expression (log norm value)
15
14
13
12
11
10
BRAF-mut
RET/PTC
RAS-like
Thyroid (healthy)

C

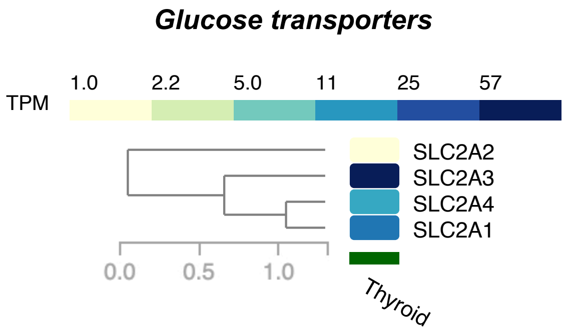

Glucose transporters
TPM
1.0
2.2
5.0
11
25
57
SLC2A2
SLC2A3
SLC2A4
SLC2A1
0.0
0.5
1.0
Thyroid

Figure S1. Differential expression pattern of metabolic genes in PTCs subtypes. (A) Graphical overview of metabolic pathways (KEGG database) enriched with genes differentially expressed between *BRAF*- and *RAS*-like PTCs in the THCA cohort. Metabolic pathways involved in lipid, carbohydrate, amino acids and nucleotide metabolism (dashed gray, orange, light purple and brown boxes, respectively) and including genes/complexes marked as $BRAF_{up}$ (blue), $BRAF_{down}$ (green), RAS_{up} (yellow), RAS_{down} (red). **(B)** Box plot displaying normalized expression values (log2 scale) of *SLC2A1* and *SLC2A3* in distinct *BRAF*-like tumor subtypes ($BRAF^{V600E}$ and *RET*/*PTC*), *RAS*-like PTCs and healthy thyroids (THCA cohort). **(C)** Heat map reporting the expression data of GTEx database for glucose transporter encoding genes.

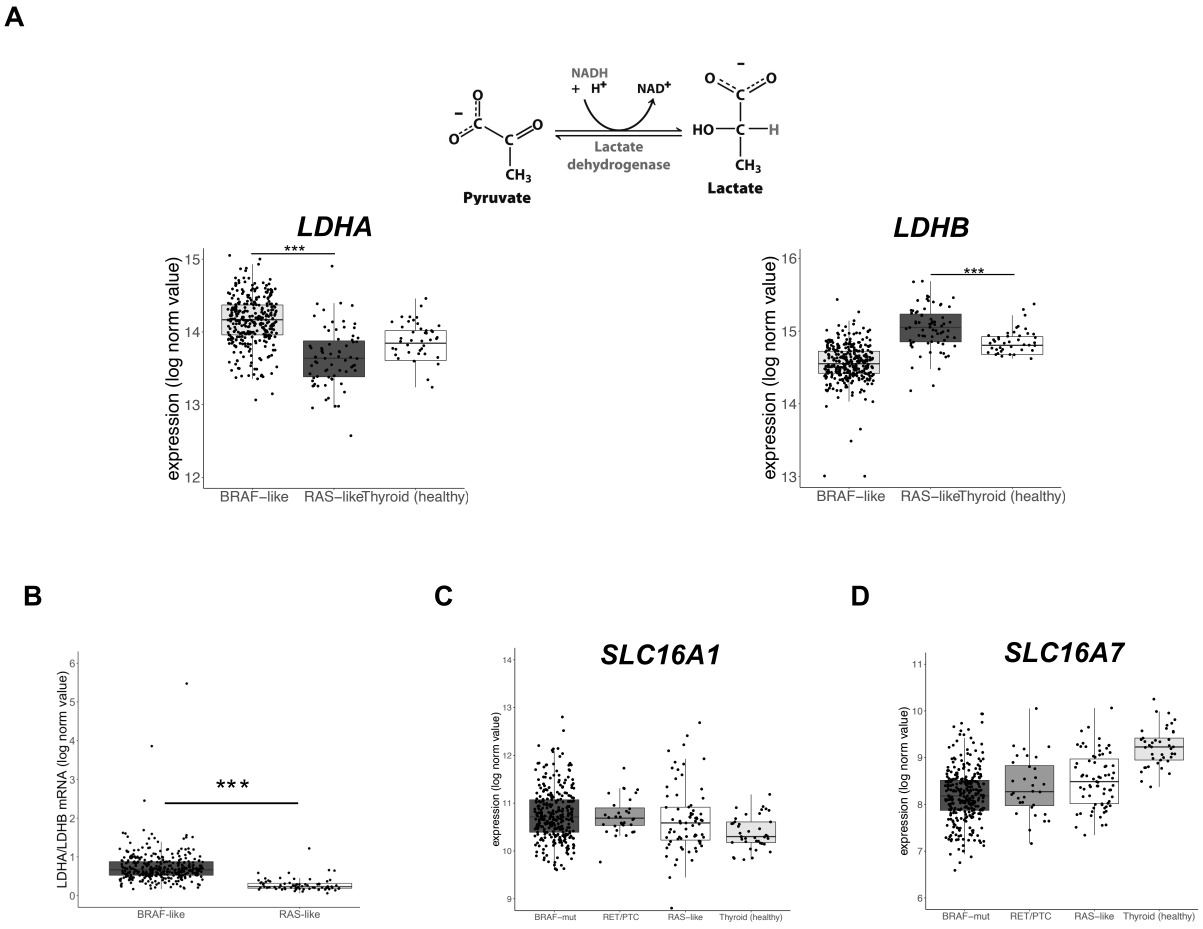
A
NADH
+ H+
NAD+
Pyruvate
Lactate dehydrogenase
Lactate
HO
CH3
LDHA
***
expression (log norm value)
BRAF−like
RAS−like
Thyroid (healthy)
LDHB
***
expression (log norm value)
BRAF−like
RAS−like
Thyroid (healthy)
B
LDHA/LDHB mRNA (log norm value)
***
BRAF-like
RAS-like
C
SLC16A1
expression (log norm value)
BRAF−mut
RET/PTC
RAS−like
Thyroid (healthy)
D
SLC16A7
expression (log norm value)
BRAF−mut
RET/PTC
RAS−like
Thyroid (healthy)

Figure S2. Differential expression of genes involved in lactate metabolism in PTCs subtypes. (A) Box plot showing normalized expression values (log_2 scale) of *LDHA* and *LDHB* in *BRAF*-, *RAS*-like PTCs and in healthy thyroids (THCA cohort), and schematic representation of the enzymatic reaction catalyzed by lactate dehydrogenase. ***FDR<0.001. **(B)** Ratio of *LDHA* and *LDHB* (*LDHA*/*LDHB*) normalized expression values (log_2 scale) in each tumor sample from *BRAF*- and *RAS*-like PTC subtypes. ***FDR<0.001. **(C-D)** Box plot displaying normalized expression values (log_2 scale) of *SLC16A1* (C) and *SLC16A7* (D) in *BRAF*-, *RAS*-like PTCs and in healthy thyroids (THCA cohort).

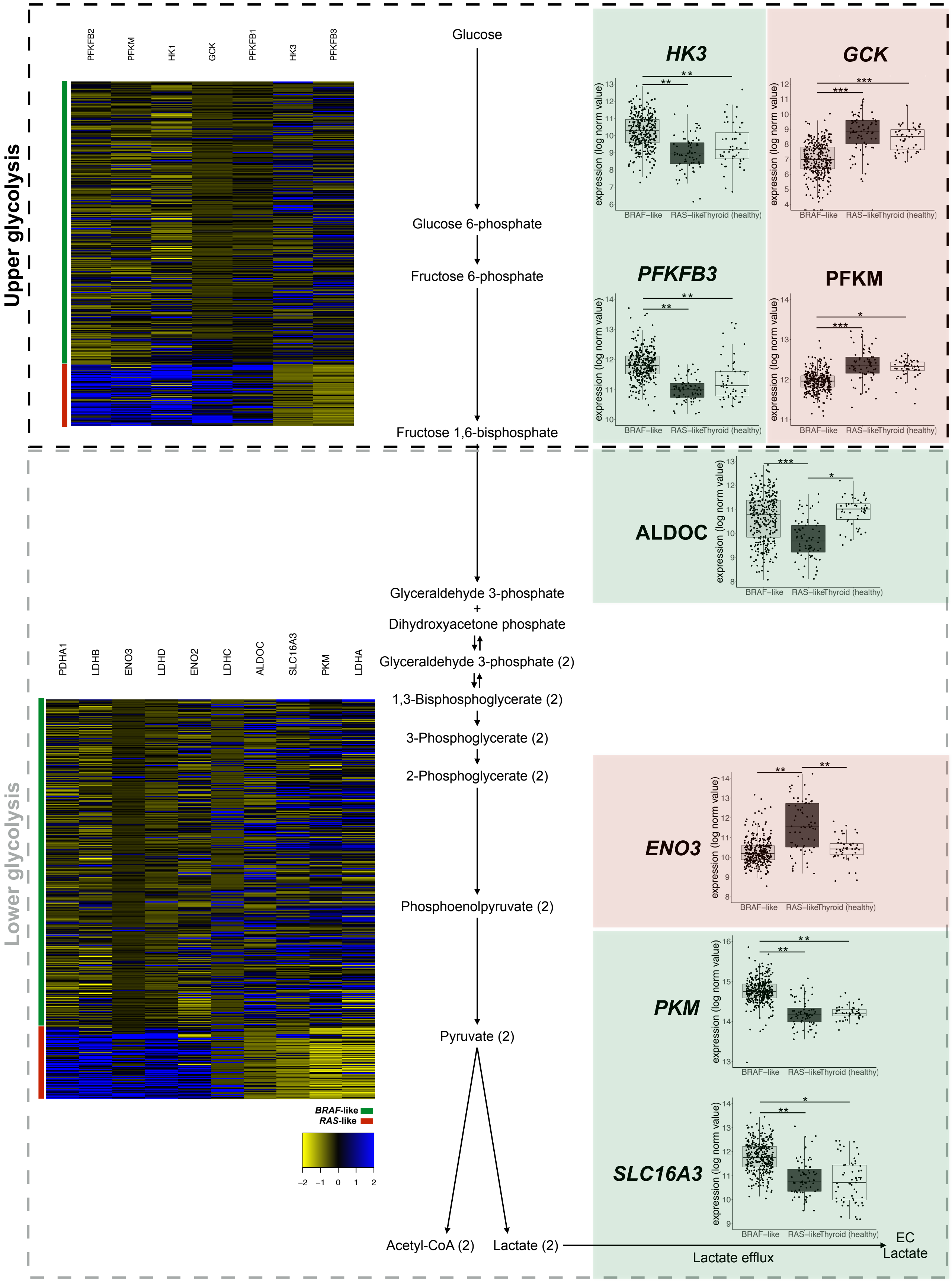

Upper glycolysis
Lower glycolysis
PFKFB2
PFKM
HK1
GCK
PFKFB1
HK3
PFKFB3
Glucose
Glucose 6-phosphate
Fructose 6-phosphate
Fructose 1,6-bisphosphate
Glyceraldehyde 3-phosphate
+
Dihydroxyacetone phosphate
Glyceraldehyde 3-phosphate (2)
1,3-Bisphosphoglycerate (2)
3-Phosphoglycerate (2)
2-Phosphoglycerate (2)
Phosphoenolpyruvate (2)
Pyruvate (2)
Acetyl-CoA (2)
Lactate (2)
Lactate efflux
EC
Lactate
HK3
GCK
PFKFB3
PFKM
ALDOC
ENO3
PKM
SLC16A3
expression (log norm value)
BRAF–like
RAS–like
Thyroid (healthy)
PDHA1
LDHB
ENO3
LDHD
ENO2
LDHC
ALDOC
SLC16A3
PKM
LDHA
BRAF-like
RAS-like
−2 −1 0 1 2

Figure S3. Differential rewiring of genes within the glycolytic flux in *BRAF*-and *RAS*-like PTCs. Upper and lower panels refer to the expression of genes encoding the enzymes involved in the upper and lower glycolysis, respectively, as displayed in the central scheme that recapitulates all the enzymatic reactions of the process. On the left, the heat maps showing normalized expression data of upper glycolysis genes in the *BRAF*- (green) *vs* *RAS*-like (red) PTCs. On the right, the box plots display expression values ($\log_2$ scale) of genes marked as $BRAF_{up}$ or RAS_{down} (highlighted by green boxes) and $BRAF_{down}$ (highlighted by red boxes). Genes defined as RAS_{up} have not been detected in glycolytic flux. *FDR≤0.05, **FDR≤0.01, ***FDR≤0.001.

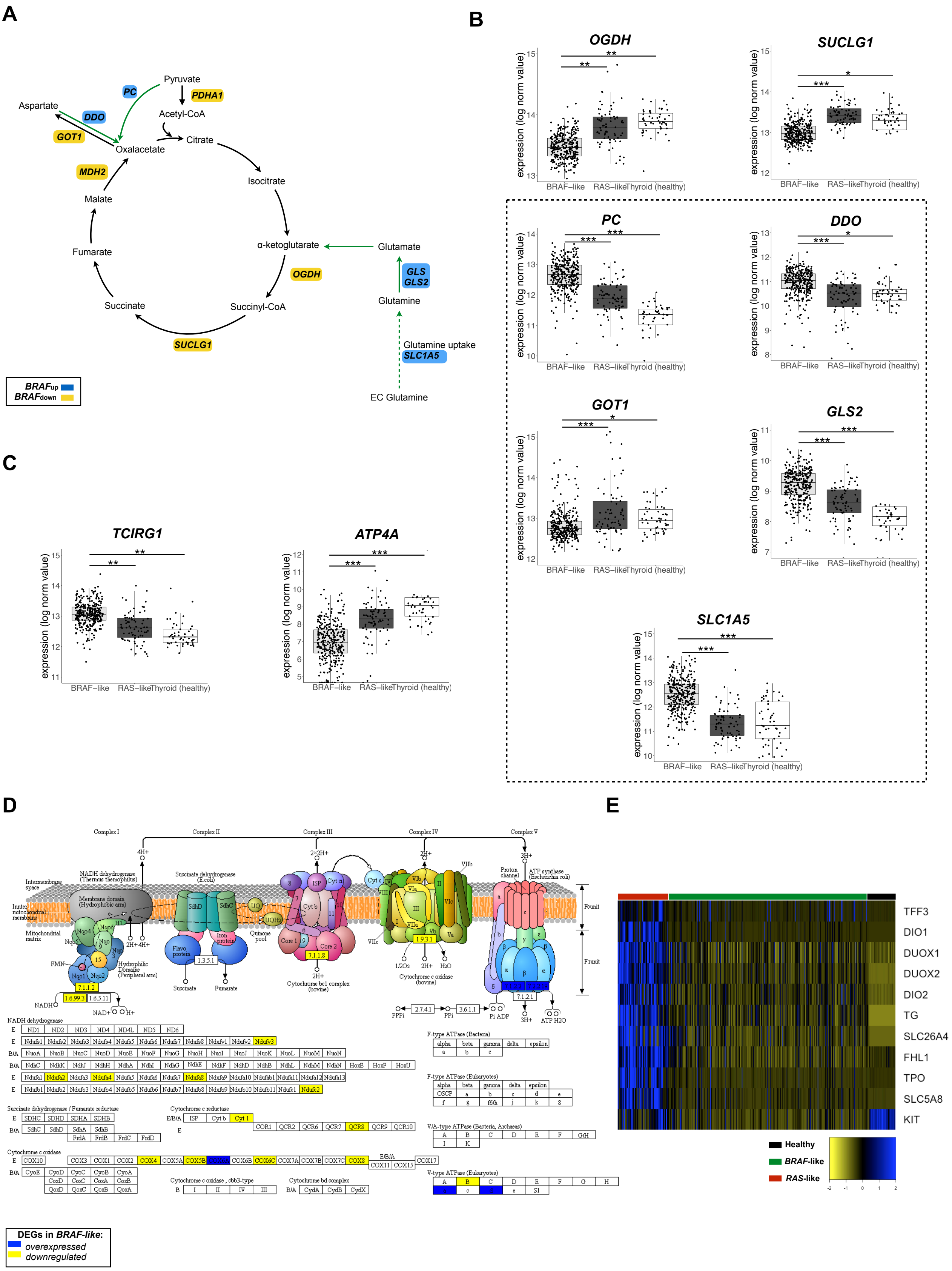
A
Pyruvate
PC
PDHA1
Acetyl-CoA
Aspartate
DDO
GOT1
Oxalacetate
Citrate
MDH2
Isocitrate
Malate
Fumarate
α-ketoglutarate
Glutamate
OGDH
GLS
GLS2
Glutamine
Succinate
Succinyl-CoA
SUCLG1
Glutamine uptake
SLC1A5
EC Glutamine
BRAFup
BRAFdown
B
OGDH
SUCLG1
PC
DDO
GOT1
GLS2
SLC1A5
expression (log norm value)
BRAF−like
RAS−like
Thyroid (healthy)
C
TCIRG1
ATP4A
D
Complex I
Complex II
Complex III
Complex IV
Complex V
NADH dehydrogenase
Succinate dehydrogenase / Fumarate reductase
Cytochrome c reductase
Cytochrome c oxidase
F-type ATPase (Bacteria)
F-type ATPase (Eukaryotes)
V/A-type ATPase (Bacteria, Archaeas)
V-type ATPase (Eukaryotes)
DEGs in BRAF-like:
overexpressed
downregulated
E
TFF3
DIO1
DUOX1
DUOX2
DIO2
TG
SLC26A4
FHL1
TPO
SLC5A8
KIT
Healthy
BRAF-like
RAS-like

Figure S4. Differential expression of metabolic genes involved in TCA, anaplerotic reactions and OXPHOS in PTCs subtypes. (A) Schematic representation of TCA cycle and related anaplerotic (green arrows) and cataplerotic reactions. Genes differentially expressed in *BRAF*- *vs RAS*-like PTCs and marked as $BRAF_{up}$ and $BRAF_{down}$ are depicted in blue and yellow, respectively. **(B)** Box plots display the expression values (log2 scale) of genes - highlighted in panel A - in *BRAF*-, *RAS*-like tumors and in healthy thyroid samples (THCA cohort). Genes encoding enzymes within the TCA cycle are shown in the upper part, whereas those responsible for the anaplerotic fluxes are reported in the dashed box below. *FDR≤0.05, **FDR≤0.01, ***FDR≤0.001. **(C)** Box plots display the expression values (log2 scale) of *ATP4A* and *TCIRG1* genes – involved in OXPHOS – in *BRAF*-, *RAS* -like PTCs and in healthy thyroid samples (THCA cohort). **FDR≤0.01, ***FDR≤0.001. **(D)** Graphical representation of ATP production via OXPHOS (KEGG pathway) showing differentially expressed genes (DEGs) overexpressed and downregulated in *BRAF*-like PTCs (highlighted in blue and in yellow, respectively). **(E)** Heatmap displaying normalized expression data of genes encoding markers of tumor aggressiveness in healthy thyroids (black), *BRAF*- (green) and *RAS*-like (red) PTCs (RNA-Seq from THCA; n=448).

A

Aminoacid biosynthesis

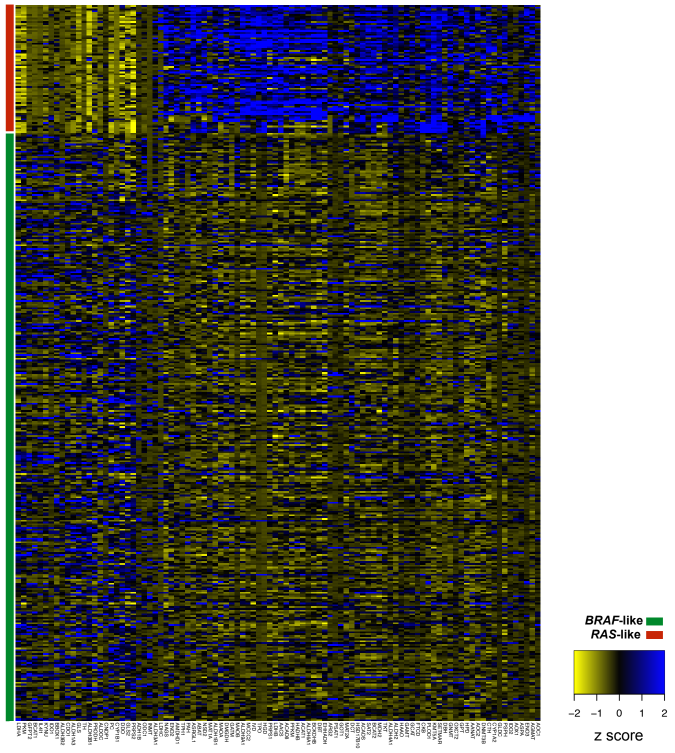

B

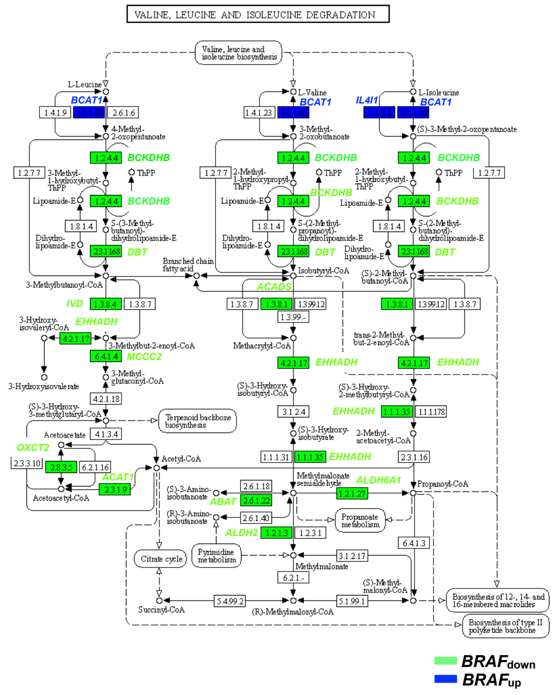

C

Lipid metabolism

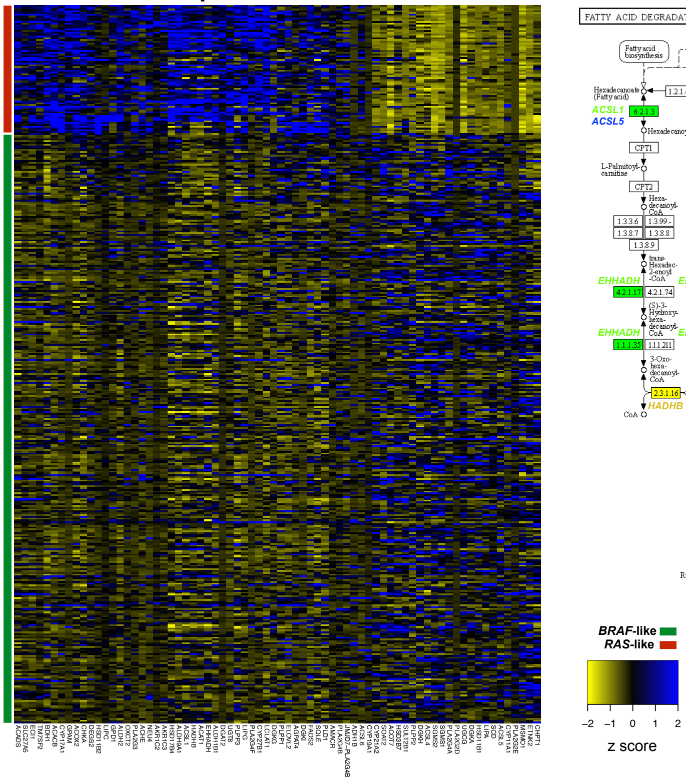

D

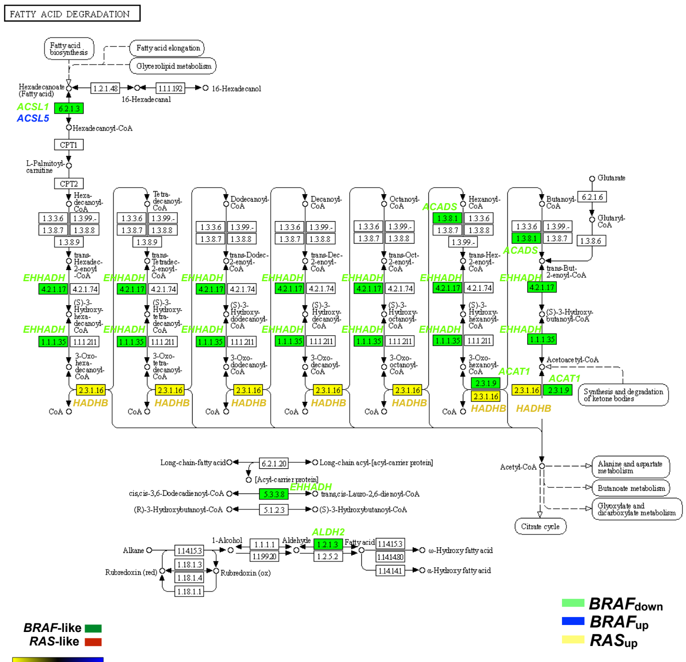

Figure S5. Differential expression of metabolic genes involved in amino acids biosynthesis and lipid metabolism in PTCs subtypes. (A) Heat map showing normalized expression data (RNA-Seq from THCA; n=398) for *BRAF*- (green) and *RAS*-like (red) PTCs for metabolic genes involved in amino acids biosynthesis, according to KEGG database classification. **(B)** Graphical representation of valine, leucine, isoleucine degradation processes, enriched with genes differentially expressed between *BRAF*- and *RAS*-like PTCs and vs healthy thyroids (THCA cohort). Genes/complexes identified as $BRAF_{up}$ and $BRAF_{down}$ are highlighted in blue and green, respectively. **(C)** Heat map showing normalized expression data (RNA-Seq from THCA; n=398) for *BRAF*- (green) and *RAS*-like (red) PTCs for metabolic genes involved in lipid metabolism, according to KEGG database classification. **(D)** Graphical representation of fatty acid degradation pathway, enriched of genes differentially expressed between *BRAF*- and *RAS*-like PTCs and *vs* healthy thyroids (THCA cohort). Genes/complexes identified as $BRAF_{up}$, $BRAF_{down}$ and RAS_{up} are highlighted in blue, green and yellow, respectively.

A

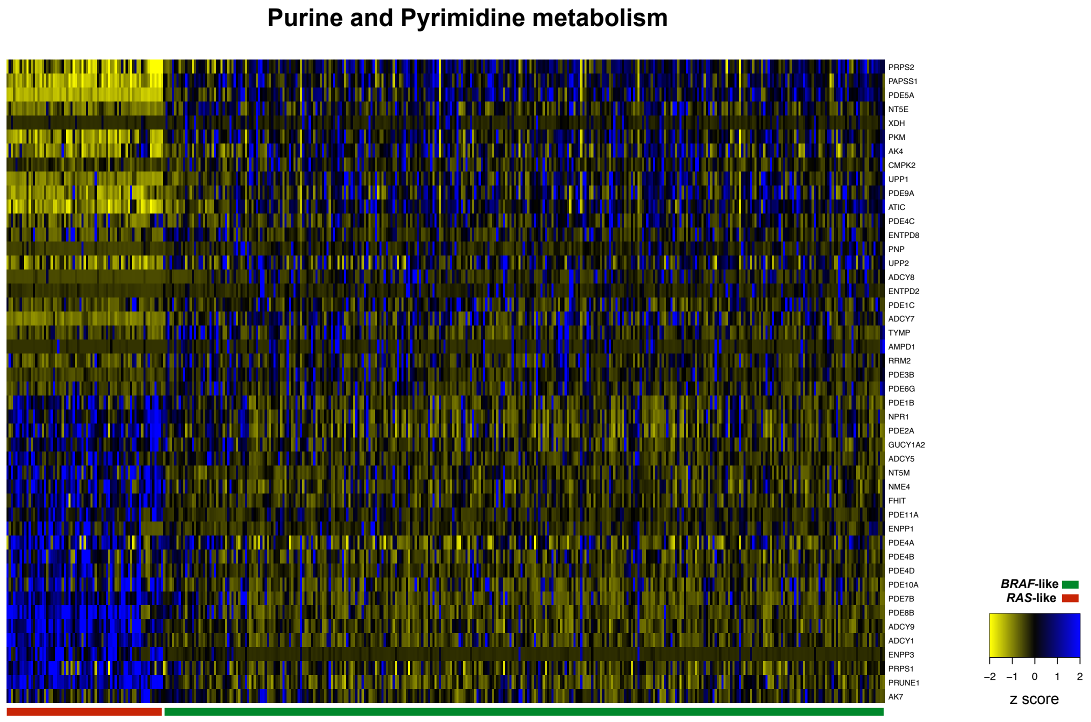
Purine and Pyrimidine metabolism
PRPS2
PAPSS1
PDE5A
NT5E
XDH
PKM
AK4
CMPK2
UPP1
PDE9A
ATIC
PDE4C
ENTPD8
PNP
UPP2
ADCY8
ENTPD2
PDE1C
ADCY7
TYMP
AMPD1
RRM2
PDE3B
PDE6G
PDE1B
NPR1
PDE2A
GUCY1A2
ADCY5
NT5M
NME4
FHIT
PDE11A
ENPP1
PDE4A
PDE4B
PDE4D
PDE10A
PDE7B
PDE8B
ADCY9
ADCY1
ENPP3
PRPS1
PRUNE1
AK7
BRAF-like
RAS-like
−2 −1 0 1 2
z score

B

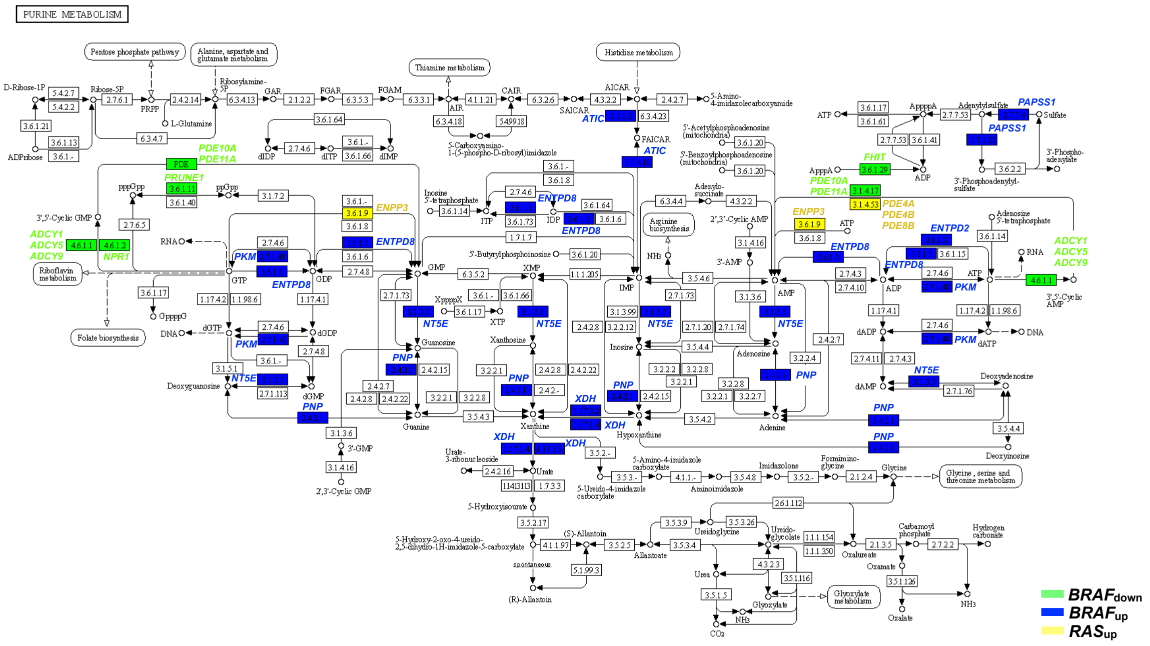
PURINE METABOLISM
Pentose phosphate pathway
Alanine, aspartate and glutamate metabolism
Thiamine metabolism
Histidine metabolism
Riboflavin metabolism
Folate biosynthesis
Arginine biosynthesis
Glycine, serine and threonine metabolism
Glyoxylate metabolism
PAPSS1
ATIC
PDE10A
PDE11A
PRUNE1
ENPP3
ENTPD8
ADCY1
ADCY5
ADCY9
NPR1
PKM
NT5E
PNP
XDH
FHIT
PDE4A
PDE4B
PDE8B
ENTPD2
BRAFdown
BRAFup
RASup

Figure S6. Differential expression of metabolic genes involved in purine and pyrimidine metabolism in PTCs subtypes. (A) Heat map showing normalized expression data (RNA-Seq from THCA; n=398) for *BRAF*- (green) and *RAS*-like (red) PTCs for metabolic genes involved in purine and pyrimidine metabolism, according to KEGG database classification. **(B)** Graphical representation of purine metabolism, enriched with genes differentially expressed between *BRAF*- and *RAS*-like PTCs and *vs* healthy thyroids (THCA cohort). Genes/complexes identified as $BRAF_{up}$, $BRAF_{down}$ and RAS_{up} are highlighted in blue, green and yellow, respectively.

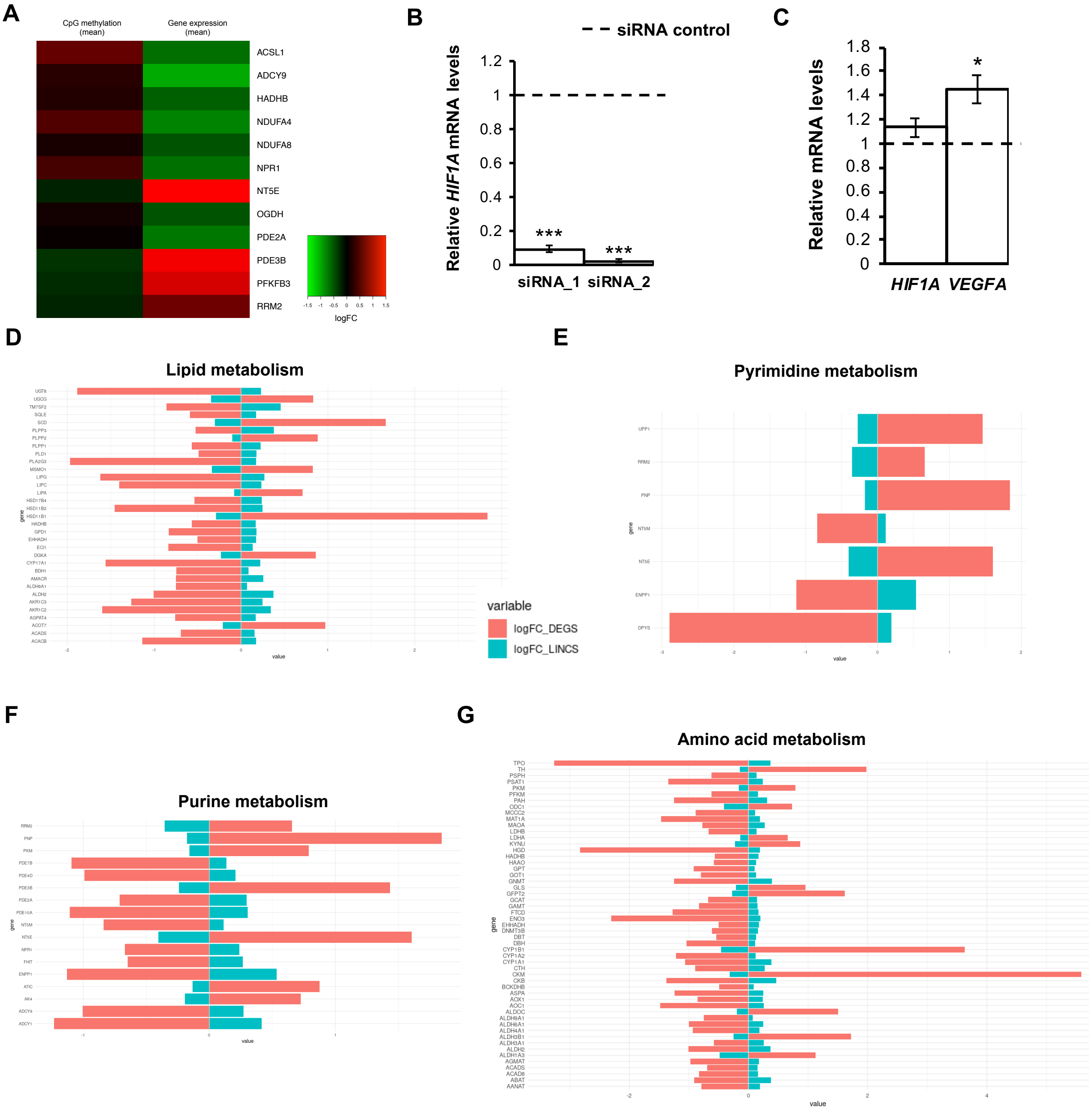
A
CpG methylation (mean)
Gene expression (mean)
ACSL1
ADCY9
HADHB
NDUFA4
NDUFA8
NPR1
NT5E
OGDH
PDE2A
PDE3B
PFKFB3
RRM2
-1.5 -1 -0.5 0 0.5 1 1.5
logFC
B
siRNA control
Relative HIF1A mRNA levels
1.2
1
0.8
0.6
0.4
0.2
0
***
***
siRNA_1 siRNA_2
C
Relative mRNA levels
1.8
1.6
1.4
1.2
1
0.8
0.6
0.4
0.2
0
*
HIF1A VEGFA
D
Lipid metabolism
variable
logFC_DEGS
logFC_LINCS
E
Pyrimidine metabolism
UPP1
RRM2
PNP
NT5M
NT5E
ENPP1
DPYS
F
Purine metabolism
G
Amino acid metabolism
gene
value

Figure S7. DNA methylation and expression correlation in metabolic genes, *HIF1A* silencing/stabilization efficacy and metabolic effects of vemurafenib. (A) Heat map showing mean expression and methylation values (logFC) for genes within clusters of genes hypermethylated and downregulated or hypomethylated and overexpressed in *BRAF*-like tumors. **(B)** Relative mRNA quantification (qPCR) of *HIF1A* expression in BCPAP transfected with two different *HIF1A* siRNAs. Data are reported as mean ±SEM vs control cells (i.e., transfected with scrambled siRNAs; dotted line) of at least three independent experiments. *PPIA* was used as reference. ***p val ≤ 0.001. **(C)** Relative mRNA quantification (qPCR) of *HIF1A* and *VEGFA* (whose over-expression has been assessed as control of CoCl2-induced Hif-1α activation) in BCPAP treated with CoCl2 (250 μM, 24h). Data are reported as mean ±SEM vs control cells (i.e., BCPAP treated with the vehicle; dotted line) of at least four independent experiments. *PPIA* was used as reference. *p val ≤ 0.05. **(D-G)** Bar graphs indicate the expression levels (logFC) of genes involved in lipid (D), pyrimidine (E), purine (F) and amino acids (G) metabolism and differentially expressed between *BRAF*- and *RAS*-like tumors, whose expression is reverted upon treatment with vemurafenib (blue bars) in multiple *BRAF*-mutated tumor cell lines from the LINCS 1000 Project. TCGA data (THCA cohort) for the same genes are indicated as red bars (logFC).

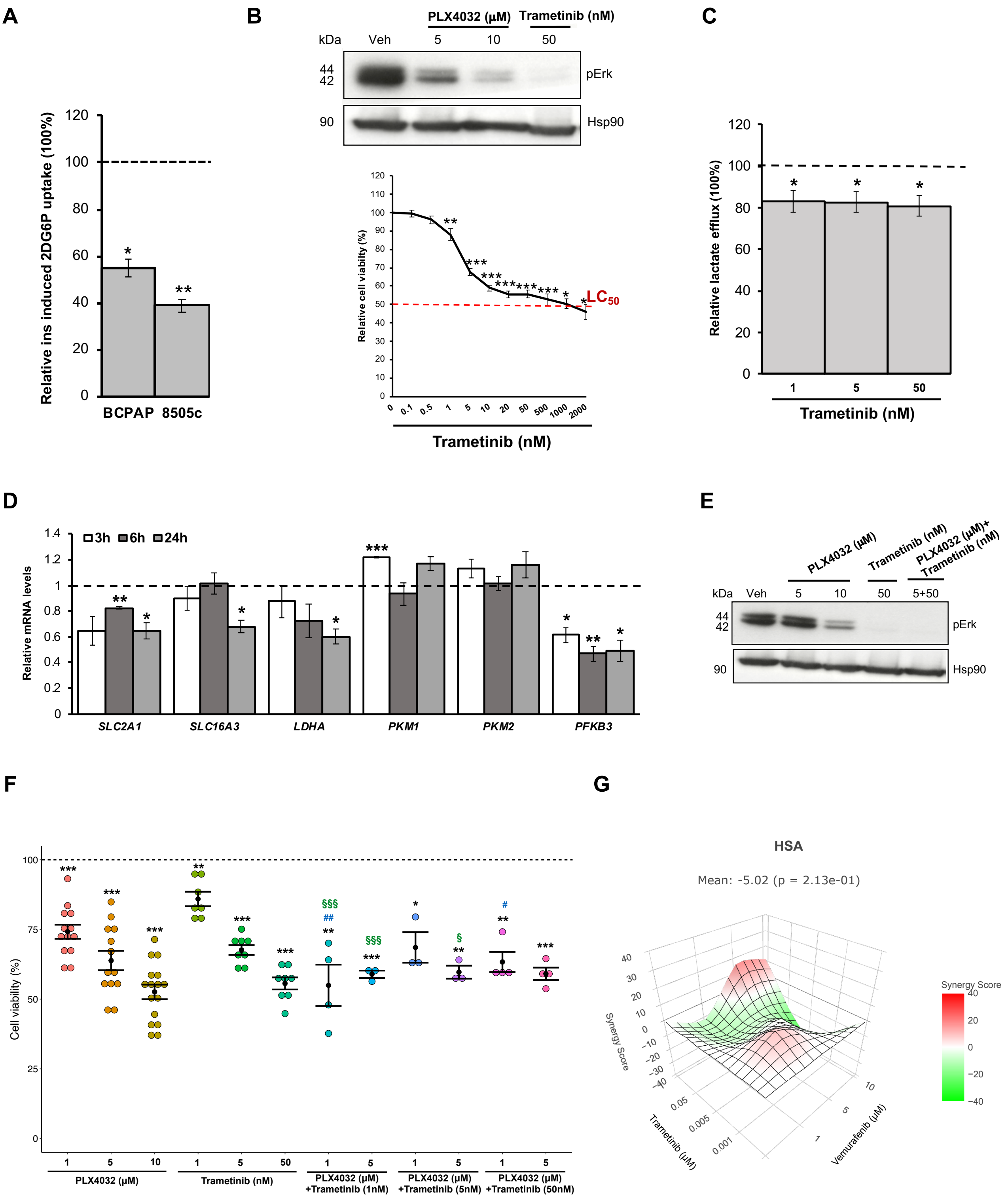

A
B
C
D
E
F
G
Relative ins induced 2DG6P uptake (100%)
BCPAP 8505c
PLX4032 (µM)
Trametinib (nM)
kDa
Veh
5
10
50
44
42
90
pErk
Hsp90
Relative cell viability (%)
LC50
Trametinib (nM)
Relative lactate efflux (100%)
Trametinib (nM)
3h 6h 24h
Relative mRNA levels
SLC2A1
SLC16A3
LDHA
PKM1
PKM2
PFKB3
PLX4032 (µM)
Trametinib (nM)
PLX4032 (µM)+ Trametinib (nM)
5+50
Cell viability (%)
PLX4032 (µM)
Trametinib (nM)
PLX4032 (µM) +Trametinib (1nM)
PLX4032 (µM) +Trametinib (5nM)
PLX4032 (µM) +Trametinib (50nM)
HSA
Mean: -5.02 (p = 2.13e-01)
Synergy Score
Trametinib (µM)
Vemurafenib (µM)

Figure S8. Trametinib modulates the expression of metabolic phenotype but does not synergize with Braf inhibitor. (A) Relative colorimetric detection of insulin-induced 2-DG6P uptake in BCPAP and 8505c treated with PLX4032 (5 μM and 10 μM, respectively) for 72h. The effect of PLX4032 treatment on insulin response was estimated as the difference between insulin-stimulated and insulin-free cells, then compared to the insulin-induced glucose uptake of control cells (i.e., cells treated with the vehicle, set to 100%; dotted line). Data are reported as mean ±SEM of three independent experiments. *p val ≤ 0.05, **p val ≤ 0.01. **(B)** Representative autoradiographs of Western blot analysis of pErk (upper panel) protein levels in 8505c treated or not with PLX4032 (5 and 10 μM) or Trametinib (50 nM) for 30 minutes. Hsp90 was used as a loading control. Relative cell viability (percentage; lower panel) in 8505c cells upon treatment with different concentrations of Trametinib (nM) for 72 hours. Red dotted line indicates the median lethal concentration (lethal concentration 50%, LC50). Data are reported as mean ±SEM *vs* control cells (i.e., 8505c treated with Veh; set to 100% of viability) in three independent experiments. *p val ≤ 0.05; **p val ≤ 0.01 and ***p val ≤ 0.001. **(C)** Relative colorimetric detection of L-lactic acid content in cell culture supernatant of 8505c treated with Trametinib (1, 5 and 50 nM) for 72 hours. Lactate concentration (mmol/L) was normalized for the related AUC and data are reported as mean ±SEM of at least four independent experiments. The effect of each treatment was estimated as the percentage of lactate secreted by control cells (i.e., 8505c treated with the vehicle, set to 100%; dotted line). *p val ≤ 0.05. **(D)** Relative mRNA quantification (qPCR) of selected metabolic genes in 8505c treated with Trametinib (50 nM) for 3, 6 and 24 hours. Data are reported as mean ±SEM *vs* control cells (i.e., treated with the vehicle; dotted line) of three independent experiments. *PPIA* was used as reference gene. *p val ≤ 0.05, **p val ≤ 0.01 and ***p val ≤ 0.001. **(E)** Representative autoradiographs of Western blot analysis of pErk protein levels in 8505c treated or not with PLX4032 (5 and 10 μM), Trametinib (50 nM) or PLX4032 and Trametinib (5μM and 50nM, respectively) combination for 30 minutes. Hsp90 was used as a loading control. **(F)** Relative cell viability in 8505c cells upon single (PLX4032 and Trametinib) and combined treatment with the two drugs at different doses for 72 hours. Data are reported as mean ±SEM *vs* control cells (i.e., 8505c treated with the vehicles - indicated as dotted line - are set to 100% of viability) of at least three independent experiments. *p val ≤ 0.05, **p val ≤ 0.01 and ***p val ≤ 0.001 *vs* control cells. $^{\#}$p val ≤ 0.05 and $^{\#\#}$p val ≤ 0.01 *vs* 8505c treated with PLX4032 alone. §p val ≤ 0.05 and §§§p val ≤ 0.001 *vs* 8505c treated with Trametinib alone. **(G)** Heat map showing in 3D the HSA synergy scores (i.e., positive values in red denote synergy), analyzed by SynergyFinder tool, for the combination of Trametinib and PLX4032 in 8505c.

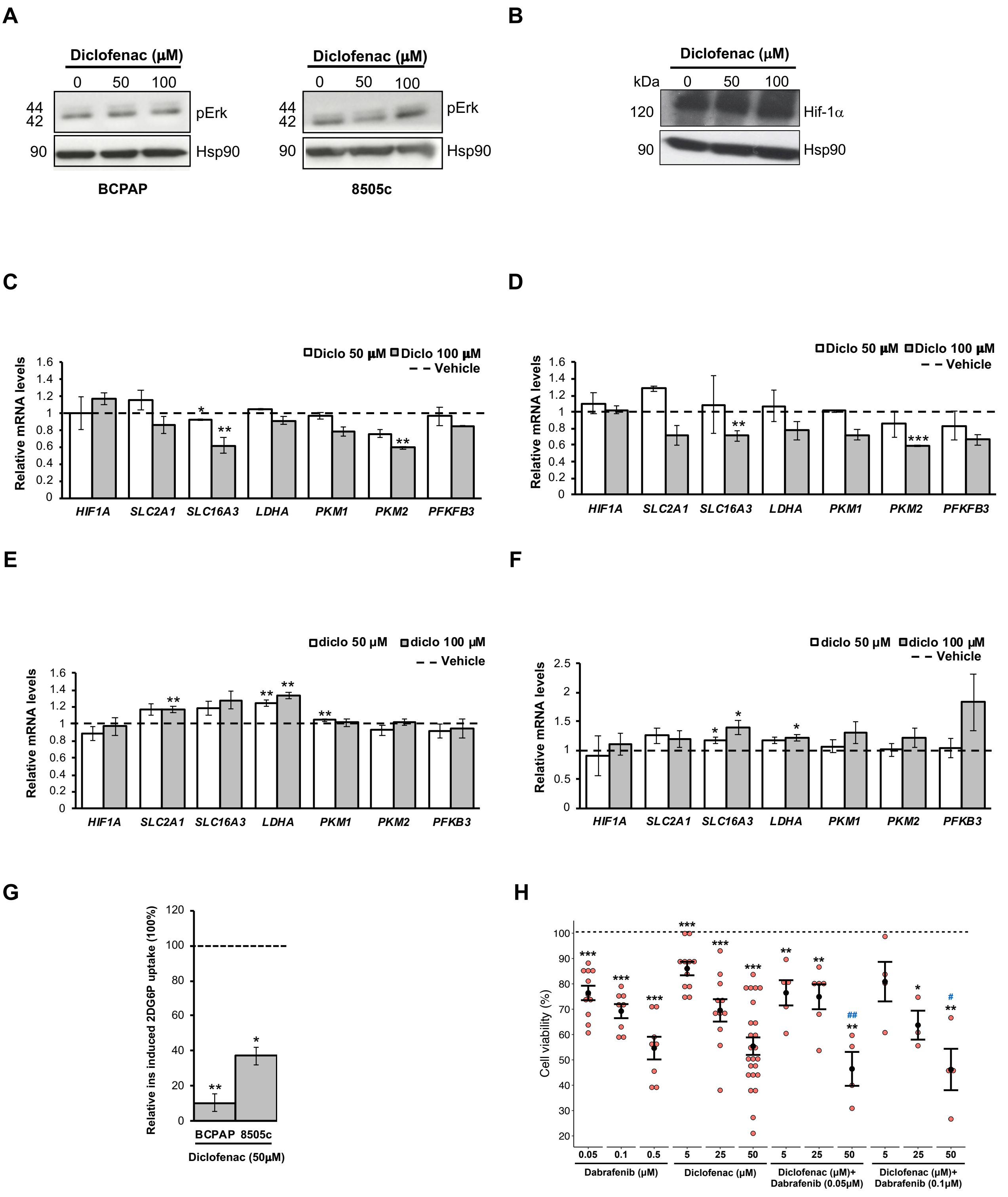
A
Diclofenac (µM)
0 50 100
44
42
pErk
90
Hsp90
BCPAP
8505c
B
Diclofenac (µM)
kDa 0 50 100
120
Hif-1α
90
Hsp90
C
Diclo 50 µM Diclo 100 µM
Vehicle
Relative mRNA levels
HIF1A SLC2A1 SLC16A3 LDHA PKM1 PKM2 PFKFB3
D
Diclo 50 µM Diclo 100 µM
Vehicle
Relative mRNA levels
HIF1A SLC2A1 SLC16A3 LDHA PKM1 PKM2 PFKFB3
E
diclo 50 µM diclo 100 µM
Vehicle
Relative mRNA levels
HIF1A SLC2A1 SLC16A3 LDHA PKM1 PKM2 PFKB3
F
diclo 50 µM diclo 100 µM
Vehicle
Relative mRNA levels
HIF1A SLC2A1 SLC16A3 LDHA PKM1 PKM2 PFKB3
G
Relative ins induced 2DG6P uptake (100%)
BCPAP 8505c
Diclofenac (50µM)
H
Cell viability (%)
0.05 0.1 0.5 5 25 50 5 25 50 5 25 50
Dabrafenib (µM)
Diclofenac (µM)
Diclofenac (µM)+ Dabrafenib (0.05µM)
Diclofenac (µM)+ Dabrafenib (0.1µM)

Figure S9. Diclofenac reduces the insulin-stimulated glucose uptake, but does not affect Hif-1α and glycolysis-related genes in *BRAF*-mutated PTC and ATC cells. (A) Representative autoradiographs of Western blot analysis of pErk protein levels in BCPAP (left panel) and 8505c (right panel) treated or not with Diclofenac (50 and 100 μM) for 24 hours. Hsp90 was used as a loading control. **(B)** Representative autoradiographs of Western blot analysis of Hif-1α protein levels in BCPAP treated with diclofenac (50 μM and 100 μM; 24h). Hsp90 was used as a loading control. **(C-F)** Relative mRNA quantification (qPCR) of selected metabolic genes in BCPAP (C, D) and 8505c (E, F) treated with diclofenac (50 μM and 100 μM) for 3h (C, E) and 6h (D, F). Data are reported as mean ±SEM *vs* control cells (i.e., cells treated with the vehicle; dotted lines) of at least three independent experiments. *PPIA* was used as reference gene. *p val ≤ 0.05, **p val ≤ 0.01 and ***p val ≤ 0.001. **(G)** Relative colorimetric detection of insulin-induced 2- DG6P uptake in BCPAP and 8505c treated with diclofenac (50 μM) for 72h. The effect of diclofenac treatment on insulin response was estimated as the difference between insulin stimulated and insulin-free cells, then compared to the insulin-induced glucose uptake of control cells (i.e., cells treated with the vehicle, set to 100%; dotted line). Data are reported as mean ±SEM of three independent experiments. *p val ≤ 0.05 and **p val ≤ 0.01. **(H)** Relative cell viability in BCPAP cells upon single (dabrafenib and diclofenac) and combined treatment with the two drugs at different doses for 72 hours. Data are reported as mean ±SEM vs control cells (i.e., BCPAP treated with the vehicles - indicated as dotted line - are set to 100% of viability) of at least three independent experiments. *p val ≤ 0.05, **p val ≤ 0.01 and ***p val ≤ 0.001 *vs* control cells. $^{\#}$p val ≤ 0.05, $^{\#\#}$p val ≤ 0.01 *vs* BCPAP treated with dabrefenib alone.

Supplementary File 1. Classification of metabolism-related genes.
Genes (official symbols; column A), are classified (in multiple sheets) according to the specific metabolic pathway they belong to (based on KEGG classification) and grouped based on their (exclusive or not) expression in BRAF- and/or RAS-like tumors (column B). Moreover, a label of their methylation status (TCGA data) is included (column C).

Supplementary File 2. Methylation status of metabolism-related genes.
Genes (official symbols; column A) are classified in multiple sheets according to the specific methylation cluster they belong to (based on expression and methylation concordance; see Methods) and are labeled according to their methylation status (TCGA data; column B).
