# Supplementary material for: Targeting metabolism by B-raf inhibitors and diclofenac restrains the viability of BRAF-mutated thyroid carcinomas with Hif-1α-mediated glycolytic phenotype
Source: Br J Cancer. 2023 May 17;129(2):249–65. doi: 10.1038/s41416-023-02282-2 (PMC10338540; doi:10.1038/s41416-023-02282-2)
Supplement: Supplementary file 4 — Supplementary Table [file 41416_2023_2282_MOESM4_ESM.docx]

**Table S1. Primer sequences of genes analyzed by qPCR**

| **Gene** | **Sense primer (5'-3')** | **Antisense primer (3'-5')** | **Melting Temperature (°C)** | **PCR product (bp)** |
| --- | --- | --- | --- | --- |
| *PPIA* | TACGGGTCCTGGCATCTTGT | GGTGATCTTCTTGCTGGTCT | 60 | 196 |
| *SLC2A1* | CTCCCTGCAGTTTGGCTAC | CACAGAGAAGGAGCCAATCA | 60 | 184 |
| *SLC16A3* | AGACCCCCCACAAGCATGA | TCATCACTGGCTTCTCCTAC | 60 | 199 |
| *LDHA* | AATGGGGGAAAGGCTGGGA | ACACCAGCAACATTCATTCCA | 60 | 108 |
| *HIF1A* | GCGGCGCGAACGACAAGAAA | GTGGCAACTGATGAGCAAGC | 60 | 126 |
| *PKM1* | AAGAAGCCCCGCCCCACT | CTGTGGAGTGACTTGAGGC | 60 | 214 |
| *PKM2* | AAGAAGCCCCGCCCCACT | CAGCCTCTGCCTCACGGG | 60 | 157 |
| *PFKFB3* | ACCAACTCCCCCACCGTCA | ATTGTCGGGGCGGAAGAAGT | 60 | 183 |
| *VEGFA* | GTGTGCCCCTGATGCGATG | CTGTAGGAAGCTCATCTCTC | 60 | 143 |
